# Stirred culture of cartilaginous microtissues promotes chondrogenic hypertrophy through exposure to intermittent shear stress

**DOI:** 10.1002/btm2.10468

**Published:** 2022-12-29

**Authors:** Niki Loverdou, Maxim Cuvelier, Gabriella Nilsson Hall, An‐Sofie Christiaens, Isaak Decoene, Kristel Bernaerts, Bart Smeets, Herman Ramon, Frank P. Luyten, Liesbet Geris, Ioannis Papantoniou

**Affiliations:** ^1^ Prometheus, Division of Skeletal Tissue Engineering KU Leuven Leuven Herestraat Belgium; ^2^ Skeletal Biology & Engineering Research Centre, Department of Development & Regeneration KU Leuven Leuven Herestraat Belgium; ^3^ Biomechanics Research Unit GIGA‐R In Silico Medicine, Université de Liege, Avenue de l'Hôpital 11—BAT 34 Liège 1 Belgium; ^4^ Biomechanics Section, KU Leuven Celestijnenlaan Leuven Belgium; ^5^ Biosystems Department MeBioS, KU Leuven Kasteelpark Arenberg Leuven Belgium; ^6^ Department of Chemical Engineering KU Leuven Celestijnenlaan Leuven Belgium; ^7^ Leuven Chem&Tech Celestijnenlaan Leuven Belgium; ^8^ Institute of Chemical Engineering Sciences, Foundation for Research and Technology‐Hellas (FORTH) Stadiou St, Platani Patras Greece

**Keywords:** bioreactor, cartilage microtissues, exometabolomics, mathematical modeling, shear stress

## Abstract

Cartilage microtissues are promising tissue modules for bottom up biofabrication of implants leading to bone defect regeneration. Hitherto, most of the protocols for the development of these cartilaginous microtissues have been carried out in static setups, however, for achieving higher scales, dynamic process needs to be investigated. In the present study, we explored the impact of suspension culture on the cartilage microtissues in a novel stirred microbioreactor system. To study the effect of the process shear stress, experiments with three different impeller velocities were carried out. Moreover, we used mathematical modeling to estimate the magnitude of shear stress on the individual microtissues during dynamic culture. Identification of appropriate mixing intensity allowed dynamic bioreactor culture of the microtissues for up to 14 days maintaining microtissue suspension. Dynamic culture did not affect microtissue viability, although lower proliferation was observed as opposed to the statically cultured ones. However, when assessing cell differentiation, gene expression values showed significant upregulation of both Indian Hedgehog (*IHH*) and collagen type X (*COLX*), well known markers of chondrogenic hypertrophy, for the dynamically cultured microtissues. Exometabolomics analysis revealed similarly distinct metabolic profiles between static and dynamic conditions. Dynamic cultured microtissues showed a higher glycolytic profile compared with the statically cultured ones while several amino acids such as proline and aspartate exhibited significant differences. Furthermore, in vivo implantations proved that microtissues cultured in dynamic conditions are functional and able to undergo endochondral ossification. Our work demonstrated a suspension differentiation process for the production of cartilaginous microtissues, revealing that shear stress resulted to an acceleration of differentiation towards hypertrophic cartilage.

## 1 INTRODUCTION

Patients with failing intrinsic tissue regeneration, such as those with complex and large bone defects in compromised biological conditions, need solutions coming from the field of tissue engineering.^1^ There is growing evidence that the implantation of engineered cartilage‐intermediate tissues results in efficient bone formation and bone defect regeneration^2^, ^3^ via endochondral ossification, a regenerative process mimicking embryonic limb development.^4^ Cartilage‐intermediate tissue implants have been engineered through the use of adult human progenitor cells from various sources such as bone marrow^5^, ^6^ and periosteum (hPDCs).^7^ This strategy has been explored in multiple tissue formats such as chondrogenically primed microaggregates,^8^ hypertrophic microtissues,^4^ hollow tubes^9^ and cell sheets^10^ which resulted in regeneration of critical size long bone defects.

In a recent study,^4^ the use of planar culture technologies through the use of non‐adherent microwells resulted in the formation of cartilaginous microtissue modules, able to self‐assemble in larger implants and regenerate large tibial defects in murine animal models. However, suspension culture of the abovementioned functional microtissues, needs to be further explored, as this will further pave the way towards the development of scaled up bioprocesses able to produce clinically‐relevant amounts of microtissue populations. Bioreactors allow for scalability, while providing capacity for real‐time monitoring and control of the culture and differentiation process of stem cells.^11^ Stirred tank reactors represent a universal, well‐established vessel type for the production of adult progenitor cells on microcarriers^12^ and human pluripotent stem cells.^13^ These systems have also been used to culture articular chondrocytes seeded on microcarriers.^14^ Several differentiation protocols for the production of differentiated induced pluripotent stem (iPS) cells in stirred tank bioreactors have been reported for different tissues including cardiac,^15^, ^16^ neural^17^, ^18^ and kidney^19^ cell types. Few studies have focused on the static suspension culture of cartilage microtissues from iPS cells^20^ or chondrocyte microtissues from bovine source in a stirred culture environment^21^ with limited process characterization. We have previously explored a microcarrier‐based stirred tank suspension process for expansion and differentiation of human adult periosteal derived progenitors with incomplete chondrogenic differentiation and maturation due to limited cell condensation on the microcarrier surface,^22^ hence there is still room to explore the potential to culture microtissues in stirred bioreactor systems.

An inherent component in dynamic suspension culture, is the effect of fluid‐generated shear stresses on the culture progenitor cells or microtissues. Currently there is lack of knowledge on the impact of process‐generated shear stresses on chondrogenic maturation towards hypertrophy in suspension cultures of cartilaginous microtissues. Furthermore, to date, in vitro studies have reported mixed results related to the impact of mechanical stress on chondrogenic differentiation with diverse mechanical regimes enhancing primary chondrocyte hypertrophy^3^ or inhibiting it during adult MSC culture.^5^ For a thorough characterization of the process environment to be made, CFD models have been developed.^23^ However, a limitation of these approaches is that they rely on bulk estimates without considering the discrete nature of these microtissue suspensions^24^ and the active (adhesive) properties of cell aggregates/microtissue structures.

In this study, we aimed at investigating suspension culture of cartilaginous microtissues and the role of fluid‐generated shear stresses on the process of chondrogenic differentiation towards hypertrophy. To carry out these experiments, we produced a microbioreactor system that provided the same volume and microtissue density as to that of the static‐planar setup. Mechanical stimulation during culture on single microtissues was characterized and quantified through simulations with advanced mathematical models representing individual microparticles instead of only the fluid component. Additionally, exometabolomics analysis was conducted to further characterize the cellular state due to the mechanical stimulation. Our work suggests that culture in suspension bioreactors is possible, and that shear stress alters the phenotypic and metabolic state of the microtissue with a rapid commitment to hypertrophy.

## 2 MATERIALS AND METHODS

### 2.1 Cell expansion

Cells of five female donors of age 14 ± 3 years old were isolated from periosteal biopsies as previously described.^25^ Briefly, the periosteal biopsies were washed and digested in type IV collagenase (440 units/mg; Invitrogen, BE) in growth medium (high‐glucose Dulbecco's modified Eagle's medium DMEM; Invitrogen, BE) supplemented with 10% fetal bovine serum (FBS; BioWhittaker, BE), and an antibiotic–antimycotic solution (100 units/ml penicillin, 100 μg/ml streptomycin, and 0.25 μg/ml amphotericin B; Invitrogen, BE). After digestion, all the donor cells were pooled together to create a cell pool. The cell pool was further expanded with a cell density of 5700 cells/cm^2^ (80% confluency per passage) in growth medium at 37 °C, 5% CO_2_ and 95% humidity. Growth medium was changed three times per week until 80% confluency when the cells were harvested with TrypLE™ Express (Life Technologies, UK) for 10 min at 37°C. All procedures were approved by the ethical committee for Human Medical Research (Katholieke Universiteit Leuven) and patients' informed consent forms were obtained (ML7861).

### 2.2 Micro‐tissue formation

Negative microwell molds were fabricated with polydimethylsiloxane (PDMS, Dow Corning Sylgard 184 elastomer, MAVOM Chemical Solutions) as described elsewhere.^26^ To create microwells (150 μm depth, 200 μm diameter), 3% agarose (Thermo Fisher) was poured over the PDMS mold and let to cool down. The agarose layer with microwells was punched into dimensions fitting tightly a 24 well plate (area ~ 1.8 cm^2^) and placed in a 24‐well plate and sterilized under UV. One 24 well plate well contained approximately 2000 microwells. Subsequently 500.000 hPDCs were seeded per well resulting in microtissues with approximately 250 cells/microtissue. Microtissues were differentiated in a xeno‐free chemically defined chondrogenic medium composed of LG‐DMEM (Gibco) supplemented with 1% antibiotic‐antimycotic (100 units/ml penicillin, 100 mg/ml streptomycin and 0.25 mg/ml amphotericin B), 1 mM ascorbate‐2 phosphate, 100 nM dexamethasone, 40 μg/ml proline, 20 μM of Rho‐kinase inhibitor Y27632 (Axon Medchem), ITS+ Premix Universal Culture Supplement (Corning) (including 6.25 μg/ml insulin, 6.25 μg/ml transferrin, 6.25 μg/ml selenious acid, 1.25 μg/ml bovine serum albumin (BSA), and 5.35 μg/ml linoleic acid), 100 ng/ml BMP‐2 (INDUCTOS), 100 ng/ml GDF5 (PeproTech), 10 ng/ml TGFβ1 (PeproTech), 1 ng/ml BMP‐6 (PeproTech) and 0.2 ng/ml FGF‐2 (R&D systems).^27^ Static controls were cultured for 16 days, and half of the media (1 ml) was exchanged 2 times per week. For the dynamic condition, cells were seeded as described earlier in order to aggregate and after 2 days microtissues were transferred to the mini‐bioreactor and cultured for an additional 14 days with 1 ml fresh medium added 2 times per week.

The small microtissues were assembled in an Aggrewell800 well culture plate to create a second static control with larger assembled microtissues (referred in the manuscript as “static large microtissues”). One well of the Aggrewell800 well plate contains an array of 300 μ‐wells with 800 μm in size. The well plate was pre‐treated using the Anti‐Adherence Rinsing Solution (STEMCELL Technologies) to reduce surface tension and prevent adhesion. Microtissues from the agarose μ‐wells (2000 microtissues) were flushed out and transferred to the AggrewellTM800 (300 microwells) 2 days after seeding. This would result in approximately seven small microtissues that fuse into a larger microtissue (Figure 2).

### 2.3 Bioreactor design and culture

The mini‐bioreactor (miniBR) setup consisted of four impeller rows, each with six impellers adapted to fit onto a 24 well plate (Corning). The impeller rows and exchangeable impellers were printed using polyamide 12, Nylon (PA12) and selective laser sintering (Formando, BE). The impeller row and impellers were connected with bevel brass gears (Reely) and metal bearings (W 604‐2RS1, SKF) to allow smooth stirring. Each row of six impellers was driven by a ST3518 stepper motor (Nanotec) driven by SMCI‐12 motor controllers (Nanotec). In this way, 24 mini‐stirred tank reactors with a volume of 2 ml were created using four impeller rows. An in‐house developed software (Windows Forms application, .NET) written in Visual Basic was used to control the four impeller rows individually. Before use, the 24 well plate was coated with Sigmacote (Sigma Aldrich) to prevent adherence of the microtissues and all components were gas sterilized (Ethylene Oxide). The bioreactor was placed in an incubator at 37 °C, 5% CO_2_ and 95% humidity to achieve appropriate cell culture conditions. Microtissues cultured for 2 days were carefully flushed out from their microwells and 2000 microtissues were added to one well of the coated 24 well plate which corresponds to the microtissue density in the static control. The impellers were inserted and started as fast as possible to avoid microtissue fusion and the mini‐BR were run at 7, 13, and 20 rad/s corresponding to low, medium, and high speed for 14 days. Chondrogenic media was changed 2 times per week.

### 2.4 Microtissue characterization

Microtissues were characterized microscopically during the differentiation process to assess microtissue size, proliferation, and viability. Cell proliferation was assessed using the Click‐iT EdU (5‐ethynyl‐2′‐deoxyuridine) Imaging Kit (Life Technologies, USA) according to the manufacturer's protocol. Briefly, microtissues were incubated with 10 μM EdU for 48 h. Next, samples were fixed in 4% paraformaldehyde (PFA) and EdU was detected with Alexa Fluor azide and Hoechst 33342 (5 μg ml^−1^) for nuclei staining followed by visualization with fluorescence microscope (Olympus IX83) and a confocal microscope (ZEISS LSM 880, Cell Imaging Core (CIC) KU Leuven) with 1 μm step size along the z‐axis. Cell viability was qualitatively evaluated using LIVE/DEAD Viability/Cytotoxicity Kit (Invitrogen) following the manufacturers description. Briefly, microtissues were rinsed in PBS and incubated for 45 min with 2 μM calcein AM and 4 μM ethidium homodimer in PBS. After removal of the staining solution, the samples were imaged in their media using a fluorescence microscope (Olympus IX83).

### 2.5 DNA quantification and gene expression analysis

Microtissues from one well were pooled together to represent one sample and lysed in 350 μl RLT buffer (Qiagen) and 3.5 μl β‐mercaptoethanol (VWR). DNA was quantified using the Qubit dsDNA HS Assay Kit (Invitrogen). Briefly, 10 μl lysed sample was diluted in 90 μl milliQ water. Next, 5 μl was added to 195 μl of working solution, vortexed and incubated for 5 minutes at room temperature. The DNA content was measured using a Qubit R Fluorometer. Next, RNA was isolated using RNeasy Mini Kit (Qiagen) whereafter RNA concentration and quality was assessed with NanoDrop 2000 (Thermo Scientific). Complementary DNA (cDNA) was synthesized with PrimeScript reagent kit (TaKaRa) followed by quantitative real‐time polymerase chain reaction (qRT‐PCR) using SYBR® Green (Life Technologies) and StepOnePlus R Real‐Time PCR System (Applied Biosystems). The heating cycle was as follows: hold at 45 °C for 2 min, at 95 °C for 30 s, followed by 40 cycles of 95 °C for 3 s and 60 °C for 20 s. Relative differences in expression were calculated using the 2^−ΔΔCt^ method normalized to the housekeeping gene Hypoxanthine‐guanine phosphoribosyltransferase 1 (HPRT1).^28^

### 2.6 Formation of microtissue‐based implants

Microtissues from two wells of a 24 well plate (containing approximately ∼2400 microtissues) cultured statically we collected on day 14. In addition, from the miniBR after14 days microtissues were assembled from two well of a 24 well plates. In both cases microtissue suspensions were dispensed in fresh wells containing in their bottom, a layer of agarose with an inverted conical 3 mm diameter well (3 mm in diameter) developed in‐house. Microtissues were allowed to sedimented for 60 min at 37°C, 5% CO_2_ and 95% humidity leading to the entrapment of the entire population of suspended microtissues in the agarose well. Subsequently chondrogenic media was added and the microtissue assemblies were let to fuse for 24 h leading to the formation of a mechanically stable mesotissue.

### 2.7 In vivo implantation analysis

A subcutaneous mouse model was used to assess the microtissues' capacity to form bone after assembly. The fused mesotissues were implanted subcutaneously in immune compromised mice (Rj:NMRInu/nu) and explants were retrieved after 4 weeks and fixed in 4% PFA. Fixated explants were scanned with nano‐computed tomography (nano‐CT) (Pheonix Nanotom M, GE Measurement and Control Solutions) for 3D visualization of mineralized tissue. Scans were performed at 60 kV, 140 μA and with diamond target, mode 0, 1 frame average, 0 image skip, 500 ms exposure time, 2400 images and a 0.1 mm aluminum filter resulting in a voxel size of 2 μm. CTAn (Bruker micro‐CT, BE) was used for image processing of mineralized tissue based on automatic Otsu segmentation and 3D visualizations of the mineralized tissue was created in CTvox (Bruker micro‐CT, BE). All procedures on animal experiments were approved by the local ethical committee for Animal Research, KULeuven. The animals were housed according to the regulations of the Animalium Leuven, KULeuven.

### 2.8 Sampling and metabolite extraction

Ιn order to avoid bias due to sampling, we used all the media for the exometabolomic analysis. Also samples were taken each time (D0, D7, D14) 2 days after the media changes. In that way, we had consistency for changes related to the refreshing of the medium. For every sample, 10 μl of 80% methanol with 2 μM d27 myristic acid was added to 990 μl of sample. Extracts were stored overnight at −80°C and were centrifuged.

### 2.9 Liquid chromatography‐mass spectrometry (LC–MS) analysis

HPLC analysis was carried out on a reversed phase ACQUITY UPLC HSS T3 C18 (1.8 μm, 2.1 × 100mm) column from WATERS on a Q Exactive Hybrid Quadrupole‐Orbitrap (Thermo Scientific) system. The mobile phase was delivered at a flow rate of 0.250 ml/min.

The mobile phase consisted of two eluents (A and B). Eluent A was composed of 10 mM TBA and 15 mM acetic acid, and eluent B was 100% Methanol. After injection of 10 μl of sample, gradient elution was performed at 37–41–100% B with linear decreases at 0–7–14–31 min. Finally, 9 min of initial conditions was applied to reequilibrate the column for further analyses.

### 2.10 Statistical analysis

All experiments were performed with at least three replicates per condition. Data were represented as individual values or box plot, if otherwise not stated. Data were compared with one‐way or two‐way ANOVA and Tukey's multiple comparisons test. Results were considered statistically different for *p* values lower than 0.05 (*p < 0.05, **p < 0.01, ***p < 0.001). Statistical analysis was performed with GraphPad Prism 9 (GraphPad Software, Inc., USA), unless otherwise stated.

### 2.11 Computational model of cartilaginous microtissues in the miniBR

To quantify the mechanical environment of the cartilaginous microtissues, we used a lattice‐free center‐based model (CBM) coupled with a computational fluid dynamics (CFD) solver. The full model details are provided in the Supplementary Information (SI) and are briefly summarized here. The CBM was used to simulate cartilaginous microtissues inside of the miniBR while the CFD solver was used to resolve the fluid flow in the reactor. In contrast to bulk estimates, this approach considers the path history of each individual tissue, providing more accurate estimates for relevant mechanical output measures when compared with bulk average estimates.

In a stirred tank with impeller radius $R_{i}$ and rotational velocity ω, the fluid Reynold number is expressed as Ref=4ρfωRi2μf, with fluid density $\rho_{f}$ and viscosity $\mu_{f}$ We assume laminar flow for Ref<2000. Given the dimensions of the vessel, see Figure 2, this holds for ω up to 20 rad/s. Under these conditions, incompressible Stokes equations can be used to model fluid dynamic in the miniBR.

In the CBM, individual microtissues are represented by deformable spherical particles with a size distribution base on microscopy measurements. Using a discrete element‐like approach, we solved the equation of motion for each particle to simulate how the micro‐tissues moved and interacted. For this, we explicitly determined the forces acting on individual particles at each time step, see supporting information (ref 2, 3m). Furthermore, based on the forces acting on the particles and local fluid velocities we estimated, for example, the magnitude of shear stress acting on the microtissues. We estimate drag and lift forces acting on the particles due to the local fluid flow by probing the local fluid velocity, pressure, and shear gradients, see supporting information. Given the low Stokes number, $\mathrm{Stk}=\frac{t_{0}v_{t}}{2R_{p}}<1$, where $t_{0}=\frac{\rho_{p}{\left(2R_{p}\right)}^{2}}{18\;\mu_{f}}$ with $v_{t}$, $R_{p}$, $\rho_{p}$ the particle terminal velocity, radius and density, micro‐tissues are expected to follow the streamlines closely and behave as stream tracers, allowing for a one‐way CFD‐CBM coupling. Furthermore, due to Rep=2ρpvtRpμf<1, Stokes drag can be assumed, providing an analytical expression for drag force. The reported shear stress was estimated by calculating the von Mises stress of each simulated microtissue, averaged over its trajectory in the miniBR, see Figure 2a.

## 3 RESULTS

### 3.1 In‐silico characterization of the dynamic process environment in mini‐bioreactors

**FIGURE 1:**
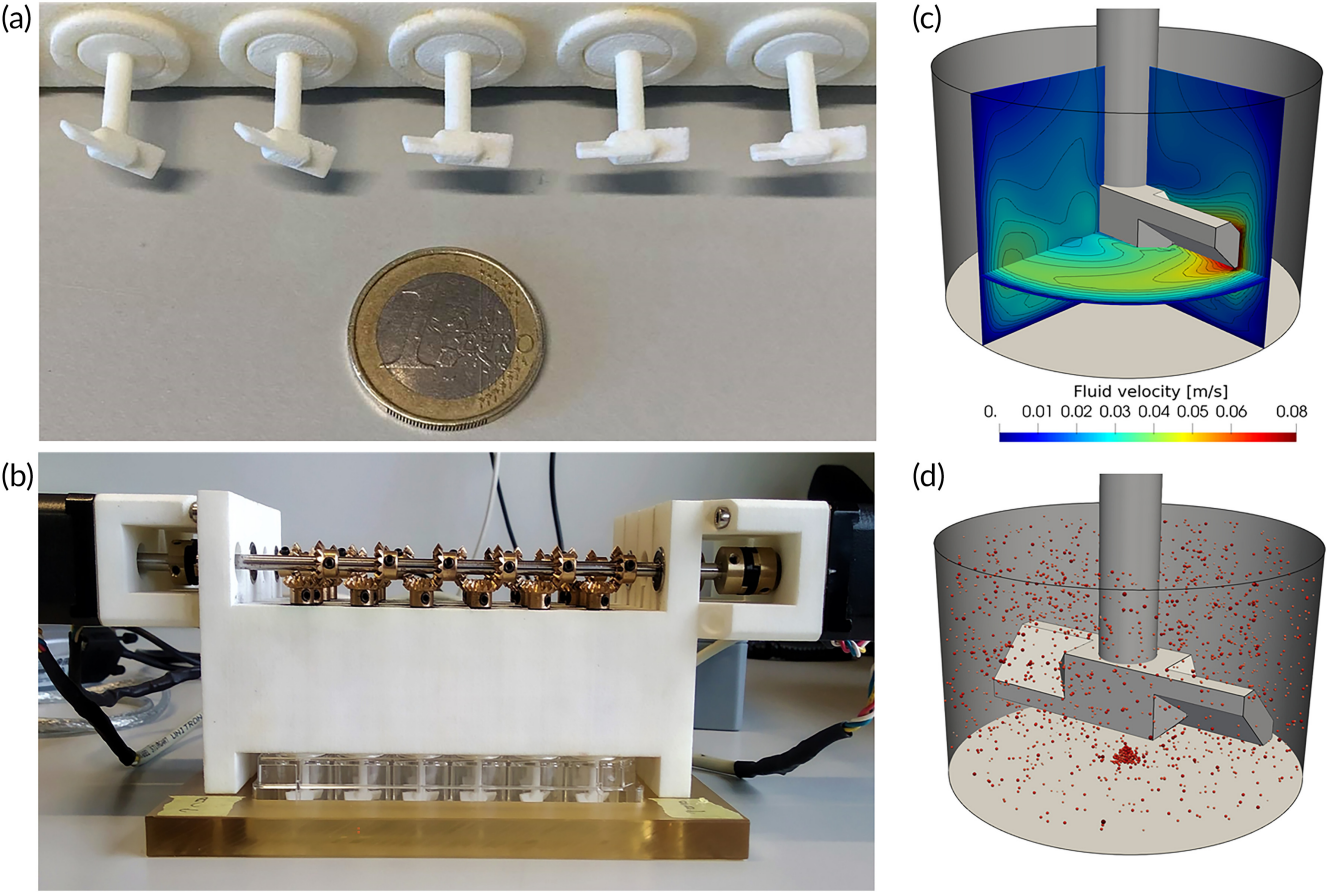
Experimental set up of the mini bioreactor and Computational Fluid Dynamics (CFD) modeling. (a, b) Mini bioreactor system with 3D printed marine impeller. (c) Cross section of CFD simulated magnitude of the fluid velocity for an impeller speed of 13 rad/s. (d) Visualization of spheroid position during stirred culture. The size of the shown spheroids has been magnified for visual clarity.

To allow high‐throughput screening of culture in stirrer‐based bioreactor culture, a mini‐bioreactor (miniBR) system was developed. Centimeter‐sized impellers were 3D printed and attached in parallel to a motor to fit commercially available 24‐well plates (Figure 1a). Four rows were produced and placed into a 24‐well plate to generate 24 stirred tank mini‐bioreactors (Figure 1b). To investigate the effect of mechanical stimulation on culture outcome, miniBR experiments with three different impeller velocities (7–13–20 rad/s), corresponding to low, medium, and high shear conditions were performed.

To estimate the magnitude of shear stress acting on the individual microtissues during dynamic culturing simulations with a coupled CBM‐CFD model were performed, see Figure 1c,d. In the computational model, we initialized particles that represent microtissues based on the observed size distribution of the dynamic culture conditions and simulated the three miniBR setups. As expected, the relative magnitude of the experienced shear stress increases with the angular velocity of the impeller. In the low shear setup, we estimated the clusters to experience shear stress in the range of 1–3 mPa, while for the high shear setup, we found a range of 6–10 mPa. The medium shear setup exhibited a broader distribution where the estimated shear stress ranged between 1 and 6 mPa.

**FIGURE 2:**
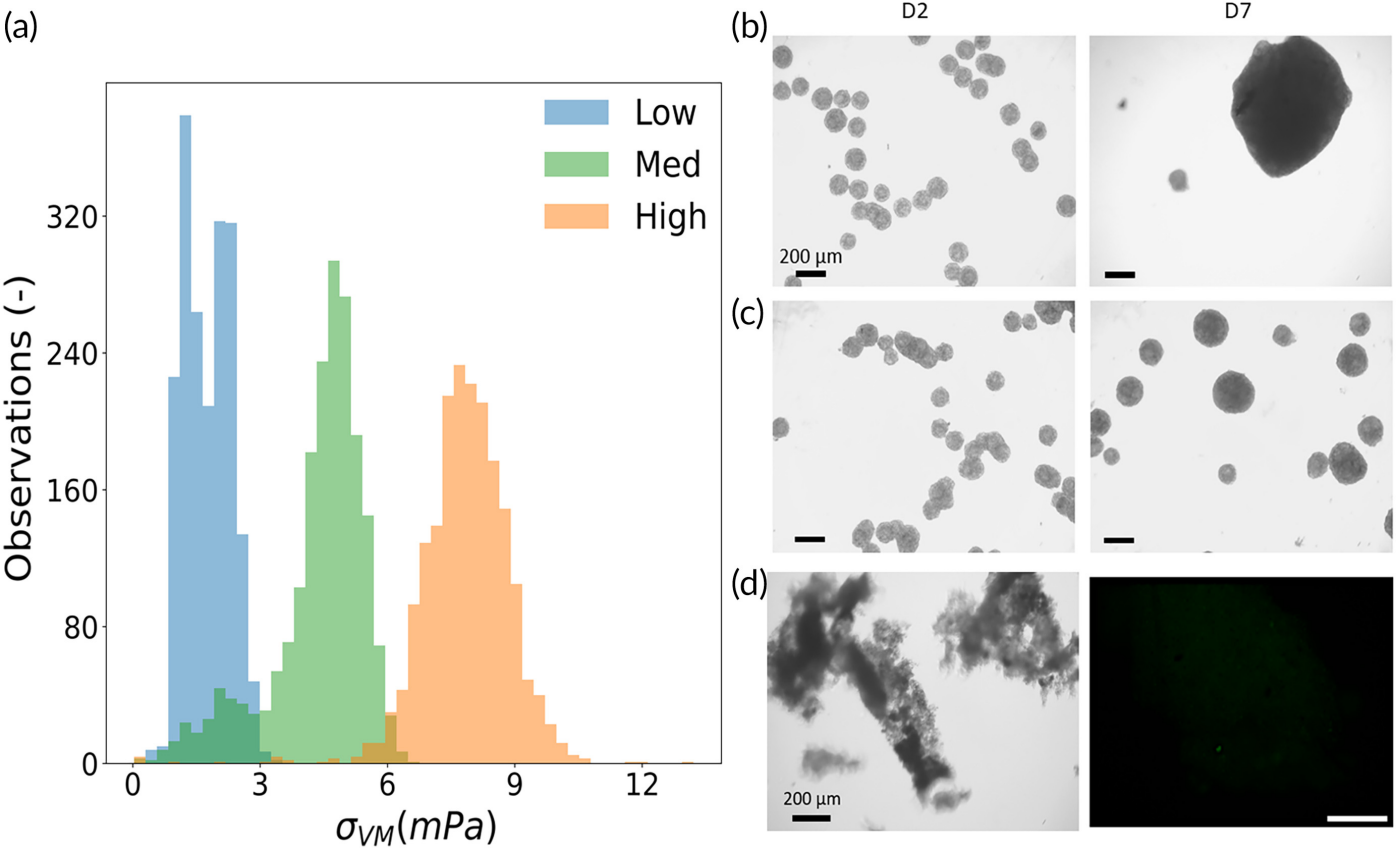
Microtissue growth after 1 week stirred culture exposed to different shear stresses. (a) Distribution of shear stress experienced by individual spheroids in low, medium, and high shear stress condition (caused by impeller speeds resp. 7, 13, and 20 rad/s). (b–d) Brightfield images of stirred cultures spheroids for Days 2 and 7 in low, medium, and high shear stress conditions. (b) The lowest rotational speed after 7 days resulted in dead zones where the microtissues were no longer suspended and merged into bigger aggregates. (c) The aggregation phenomenon also occurred in the medium speed, but smaller aggregates could also be observed. (d) At the highest rotational speed, highest hydrodynamic forces caused cell lysis or apoptosis (cell debris present already at Day 2, almost no microtissues at Day 7). Scale bar: 200 μm.

A low shear zone exists beneath the impeller while a higher shear zone was observed above the impeller. This low shear zone may cause issues for the low rpm setups, as the probability for progressive microtissue agglomeration increases in this stagnant region. For the low rpm setup, the typical shear stress in the stagnant and recirculating populations almost coincide, see Figure 2a. In the medium shear regime, the stagnant and recirculating region are associated with distinct levels of shear stress, resulting in a broader overall distribution of shear stress, see Figure 1d. We found that the stagnant region persists in a range of relative spheroid mass density $\left(\rho_{p}-\rho_{f}\right)/\rho_{f}$ between 0 and 0.06.

### 3.2 Cartilaginous differentiation in dynamic and static culture environments

**FIGURE 3:**
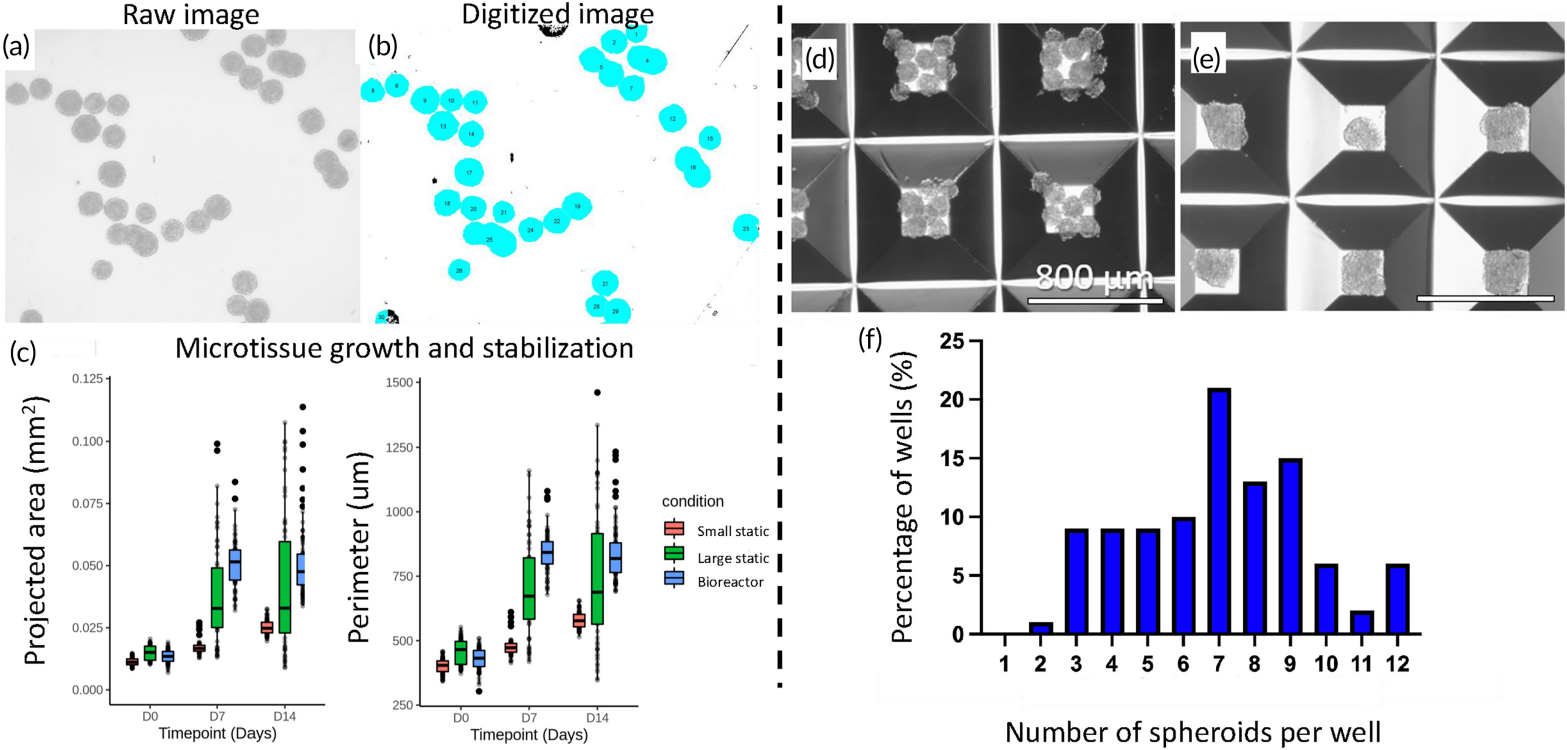
Size distribution of the stirred cultured microtissues and their static references. (a, b) Representative brightfield microscopy image of microtissues cultured in suspension were analyzed using ImageJ software through digitized images. (c) Microtissue growth results as measured by projected area and perimeter were visualized using box plots. In these box plots, the middle line indicates the median value, the box indicates the interquartile range (50% of the data), the end of the whiskers represent one and a half times the interquartile range and the dots represent outliers. (d) As a static control capturing microtissue fusion Aggrewells were used for the seeding of small microtissues. (e, f) As a second static control, multiple spheroids were seeded in larger size microwells, allowing them to fuse into larger sized microtissues. Impeller speed used is 13 rad/s. Scale bar: 800 μm

**FIGURE 4:**
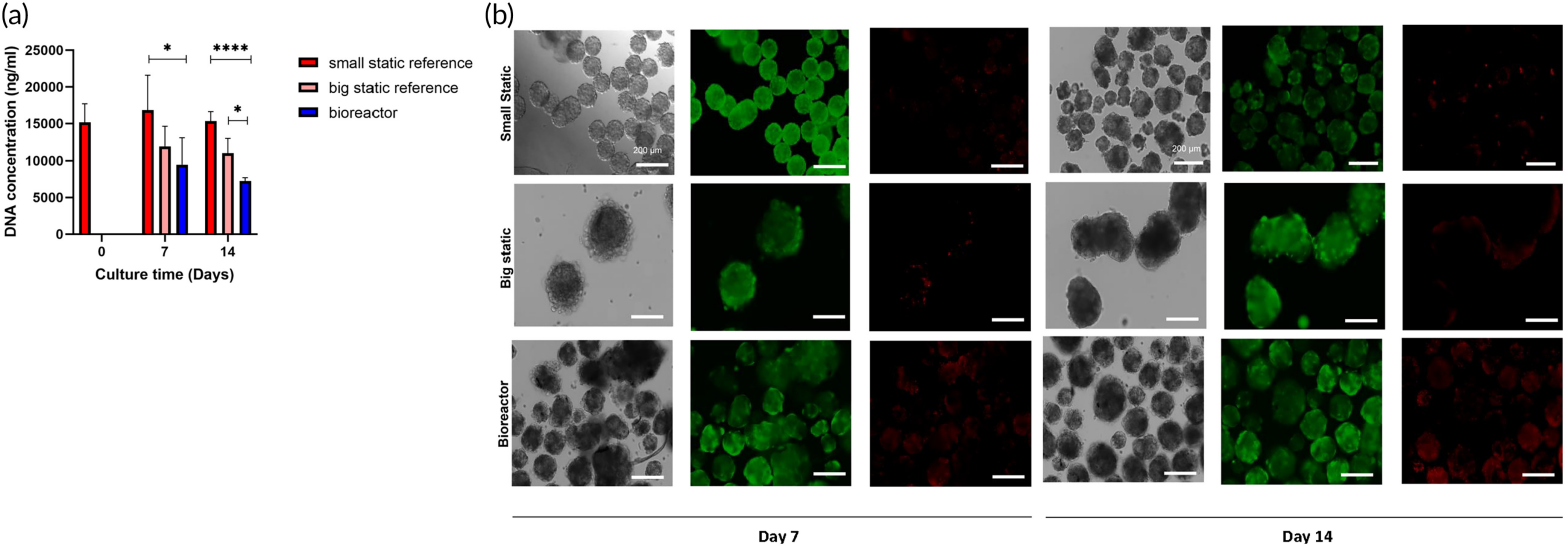
Cell viability for Days 7 and 14 by means of brightfield images (a, d), (a) DNA quantification of static and dynamic cultured microtissues over time (*n* = 4, mean values ± SEM). (b) Live staining and Dead staining. The rows respectively show results for microtissues from the small static condition (up row), large static condition (middle row) and dynamic conditions (down row). Impeller speed used is 13 rad/s. Scale bar: 200 μm

**FIGURE 5:**
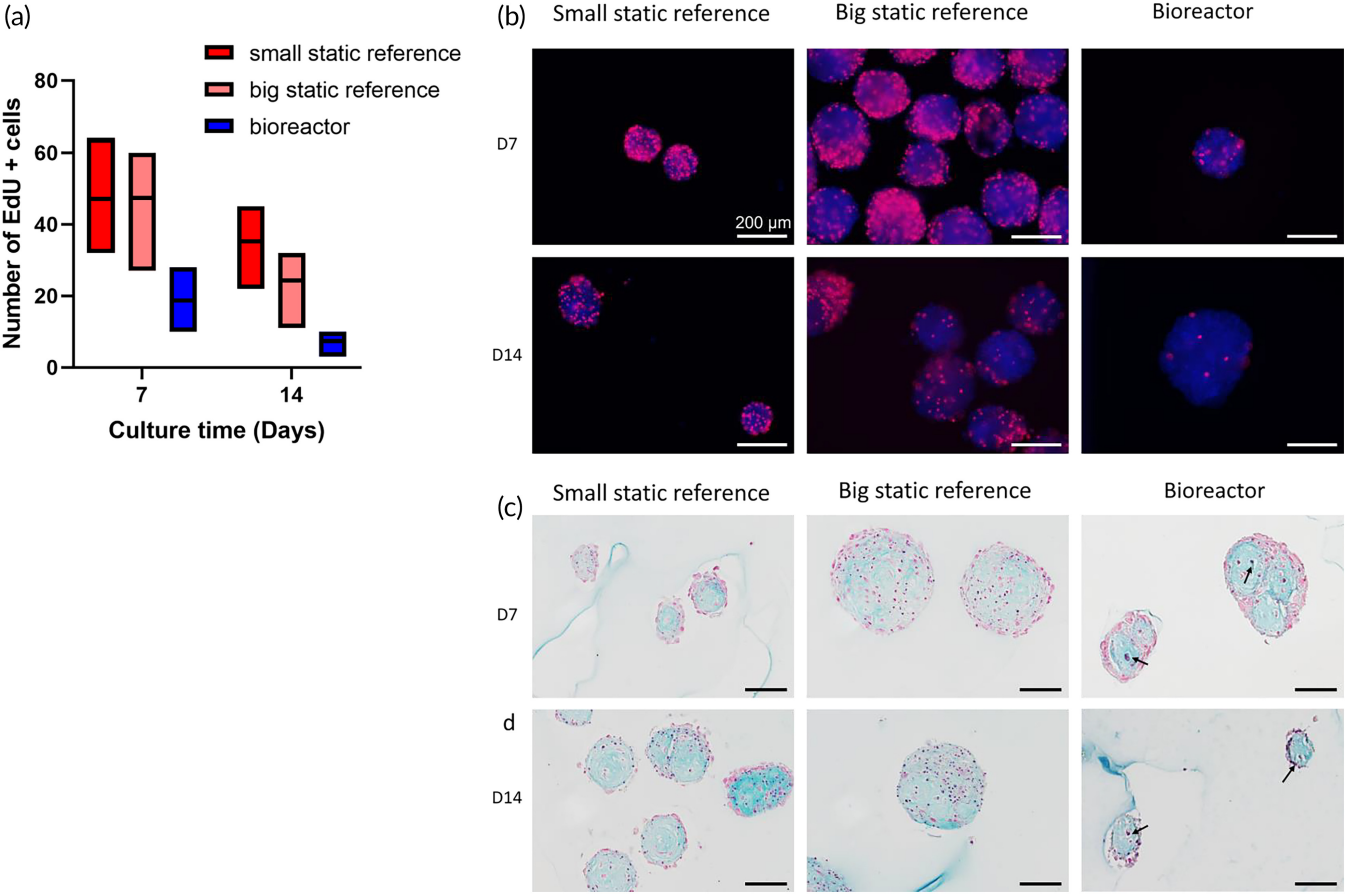
Histological assessment of microtissues in static and bioreactor conditions. (a) Semi quantification of cell proliferation of static and dynamic microtissues over time (minimum of 50 microtissues per condition). (b)Representative fluorescence images of proliferating cells (EdU positive, red) of small, bigger static and bioreactor cultured microtissues at Day 7. (c) EdU positive staining of small, bigger static and bioreactor cultured microtissues at Day 14. (d) Alcian blue staining of small, bigger static and bioreactor cultured microtissues at Day 7. In dynamic conditions, we observed fewer EdU positive cells at both Days 7 and 14. In all conditions abundant ECM compartments were present. Impeller speed used is 13 rad/s. Scale bar: 200 μm.

**FIGURE 6:**
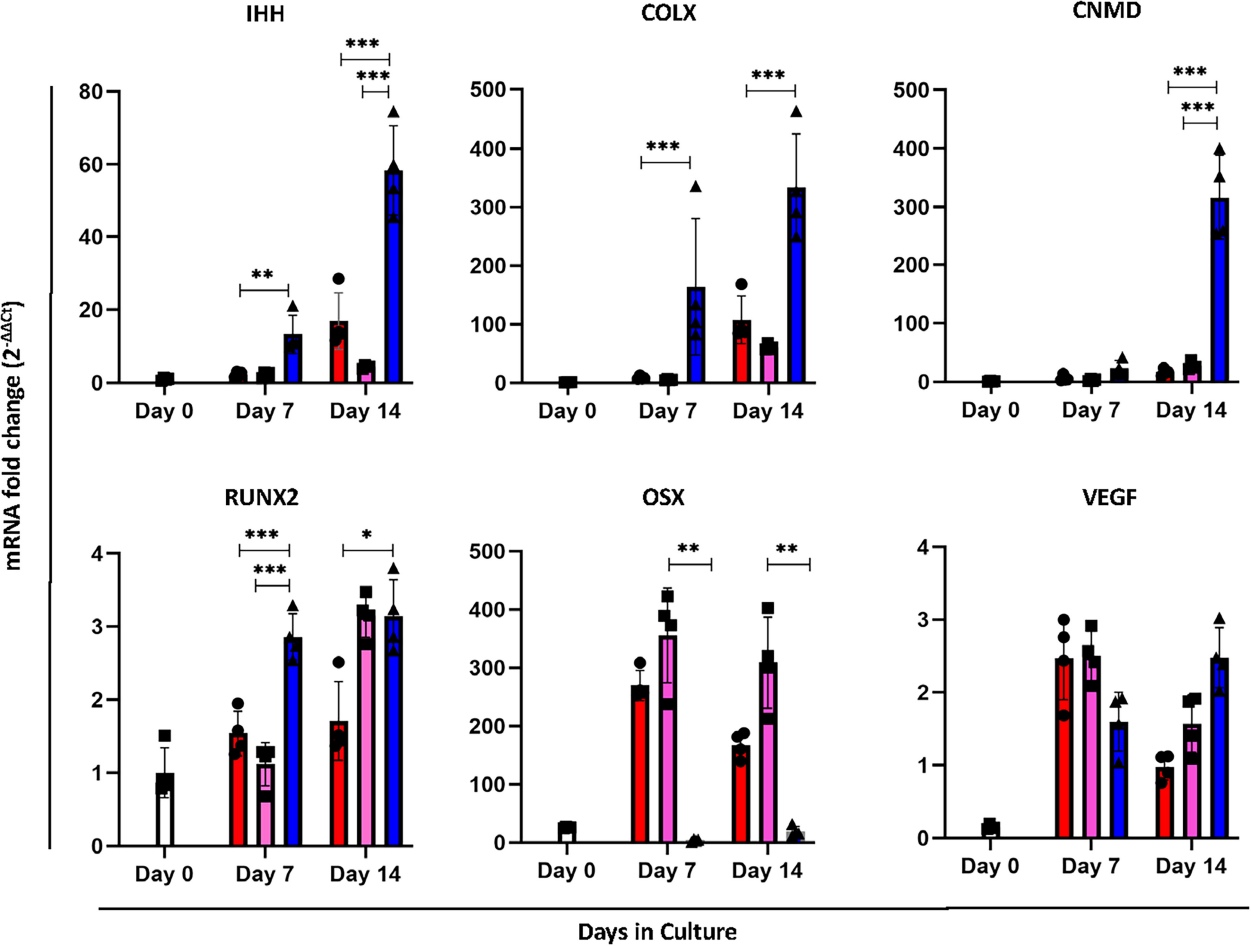
Gene expression of static and stirred cultures showed enhanced hypertrophy. Quantification of mRNA transcripts of gene markers normalized to Day 0 (*n* = 4 individual values ± SEM), *p < 0.05, **p < 0.01, ***p < 0.001, one way ANOVA followed by Tukey's multiple comparison test. Blue color represents the dynamic condition, pink the static big microtissues and red the static small microtissues. Impeller speed used is 13 rad/s.

In order to generate cartilaginous microtissues, we first allowed cells to aggregate for 2 days, forming stable microtissues, before inoculating the bioreactors. This provided a population of microtissues that possessed an almost monomodal size distribution with an average diameter of *d* = 120 μm, providing a homogeneous in size starting population. Preliminary experiments with the miniBR setup illustrated that we could culture cells dynamically to form microaggregates for up to 14 days in culture media containing chondrogenic induction factors. Furthermore, we observed that microtissues were able to fuse into larger structures during the first 3 days of culture, after which this process stopped, resulting in a steady state size distribution for the remainder of culture. This size ranged between 0.01 and 0.04 mm^2^ (projected area) as observed. To account for the phenomenon of fusion, we used two static controls; one where spheroids cultured with the same dimensions as the initial inoculation population (200–250 mm) and a second control where multiple microtissues were seeded in larger size microwells, allowing them to fuse into larger sized microtissues (400–450 mm) at the same time point as we inoculated the bioreactor (Figure 3). We quantified certain morphological attributes (Figures 3c and S1) for each microtissue population. Starting with the microtissue perimeter we noticed variation in the dynamic condition in comparison to the static references with clear difference already at Day 7 but even more distinct at Day 14. As confirmed by our observations in bright field images, bioreactor cultured microtissues were able to assemble in larger microtissues (fusion phenomenon). Some variation in the perimeter size was observed also in the static large microtissues in comparison to the small static reference as indeed in the larger microtissues there was a variation in sizes, as a result of fusion (limited in comparison to the bioreactor). Microtissue roundness in static and dynamic cultured microtissues seem to display similar results between them and across time (Figure S1). However, at Day 14 there is bigger variation for bioreactor cultured microtissues with following the large static ones, something that can be explained also with the different degree of fusion that we noticed in bioreactor cultured microtissues. Regarding microtissue circularity, we observed bigger variation in bioreactor and large static microtissues at Days 7 and 14, aligned with microtissue perimeter and microtissue roundness results. We estimated approximately the cell density in different conditions as the cell number of the microtissue divided by the microtissue volume (Figure S2). Cell density is significantly lower in dynamic and big static microtissues in comparison to the small static ones at Day 7 and the difference is even bigger at Day 14. Regarding the culture time dependent differences at Days 7 and 14 we observed a significant decrease in comparison to the starting point, Day 0 for all the conditions. There is a clear decrease in cell number also for the static condition (extrapolated by DNA quantification, Figure 4a). However, in the case of big static and bioreactor conditions apart from the significantly higher decrease we have in parallel an increase in diameter which results in significantly lower cell density. We observed that viability was not affected either by the fusion event on Day 2 or the size of the microtissues in the static controls, while the same applied for the microtissues cultured in the bioreactor which were observed to consist of a largely viable population (Figure 4 live/dead staining). In order to provide quantitative data related to cell viability and proliferation, we conducted DNA quantification (*n* = 4) for the microtissues of different conditions (small static, big static and dynamic conditions) over time. At Day 7, we observed statistically significant decrease (*p* = 0.0499) in DNA content between small static and bioreactor condition while there was a decrease but not statistically significant for DNA content between big static reference and bioreactor condition. At Day 14 there is an even larger statistically significant difference in DNA content between small static and bioreactor conditions (*p* < 0.0001). Between big static reference and bioreactor cultured microtissues, we noticed a statistically significant decrease in DNA content (*p* = 0.0135) (Figure 4a). When investigating the presence of proliferating cells, we did see a distinct difference between the static and dynamic conditions, with a lower fraction of EDU+ cells present in the bioreactor condition (Figure 5). In order to provide quantitative data related to cell proliferation, we conducted quantification of EdU positivity from fluorescent images for the different conditions and different time points. For each condition and time point, we focused on 50–200 microtissues. There is a clear decrease in EdU positive cells in the bioreactor condition for both Days 7 and Day 14 with the Day 14 showing the lower fraction of EdU positive cells (Figure 5). Small and large static microtissues display similar proliferation for Day 7, whereas there is a difference in proliferating cells at Day 14 with large static microtissues having fewer proliferative cells. These data are aligned with the DNA content quantification (Figure 4a) and suggest lower proliferation in dynamic cultured microtissues compared with the static ones. Alcian Blue staining at low pH, specific for glycosaminoglycan (GAG) confirmed the presence of cartilage‐like extracellular matrix compartments within the microtissues across conditions. We noticed an increase at Day 14 compared with Day 7 for both static and dynamic conditions. Pre‐hypertrophic like cells were visible after 2 weeks of culture (Figure 5c,d, black arrows). Gene expression values (Figure 6) showed a significant upregulation of both Indian hedgehog (*IHH*, 30‐fold) and Collagen type X (*COLX*, 23‐fold), a well know marker of chondrogenic hypertrophy, for the day 7 and day 14 time points for the dynamically cultured microtissues. Moreover, the chondrogenic marker Chondromodulin showed a large upregulation (16‐fold) for the last time point (day14). For the transcription factor *RUNX2*, linked with chondrocyte hypertrophy and early osteoblast differentiation, no statistically significant differences were seen on day14 between the static large microtissues and the dynamically cultured ones although a clear upregulation for the dynamically cultured spheroids was observed at the early timepoint (day7). The transcription factor Osterix (OSX) which is directly regulated by RUNX2 and expressed in prehypetrophic chondrocytes and osteoblasts showed a significant downregulation (0.06‐fold) for Day 14 and (0.04‐fold) for Day 7 in dynamic cultured microtissues compared with the static ones. Finally, we did not observe any statistically significant difference in the expression of VEGF between static and dynamic cultured microtissues. The VEGF expression for both static references (small and large static microtissues) follow a similar trend. At Day 7, we have a significant upregulation in comparison to Day 0 microtissues whereas at Day 14 we observed downregulation. In contrast, dynamic cultured microtissues showed an upregulation in Day 14 in comparison to Day 7.

**FIGURE 7:**
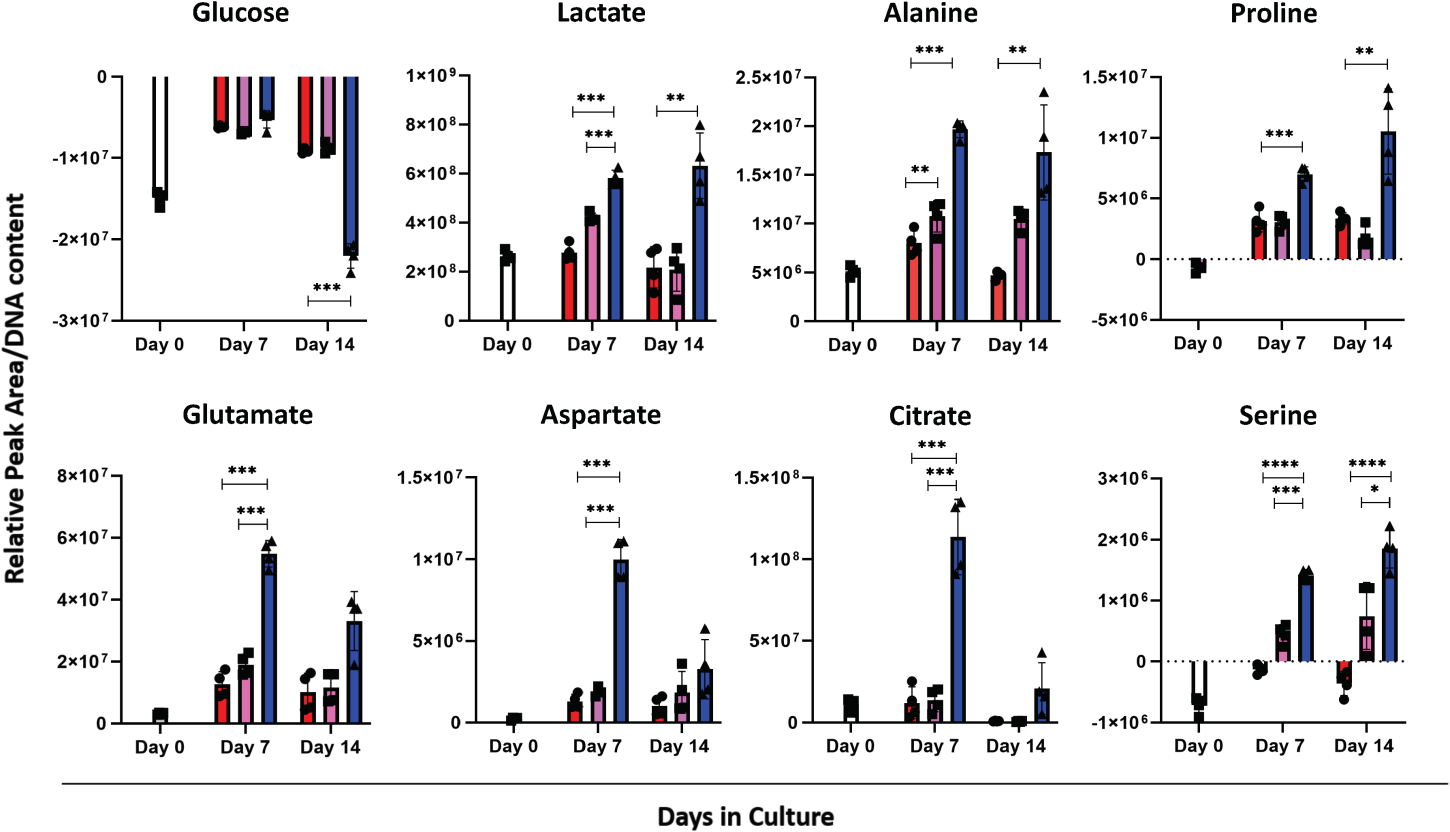
Assembly of cartilage microtissue intermediates into larger bone forming constructs for static and dynamic cultured microtissues. (a) Schematic representation of culture, assembly of microtissues and implantation in mice models. (b) Brightfield images of microtissues differentiated for 14 days either statically or dynamically in the miniBR (Day 14) and brightfield images of microtissues assembled into millimeter sized constructs (Assembled [Day14 + 1]). (c) 4 weeks subcutaneous implantation of assembled microtissues differentiated in static or dynamic (miniBR) condition. Images show 3D representation of the entire mineralized tissue and a cross section of the volume. Impeller speed of the bioreactor cultured microtissues is 13 rad/s. Scale bars represent 100 μm.

Since genes related with chondrocyte hypertrophy and the presence of prehypetrophic cells (Figure 5c,d) were detected, subcutaneous in vivo implantations were performed after 14‐days differentiation to assess the microtissues' capacity to mineralize upon implantation and execute cartilage to bone transition (i.e endochondral ossification). Approximately 2400 microtissues were assembled in an agarose macro‐well with a diameter of 3 mm, to create millimeter sized constructs as previously described^4^ (Figure 7). Constructs assembled from day 14 microtissues from both static and dynamically cultured microtissues resulted in the formation of mineralized constructs with 26 ± 14% (4/4) and 24 ± 6% (2/3) mineralized tissue (MV/TV), respectively (Figure 7) with cortical‐like bone structures.

### 3.3 Exometabolomics of chondrogenic differentiation

**FIGURE 8:**
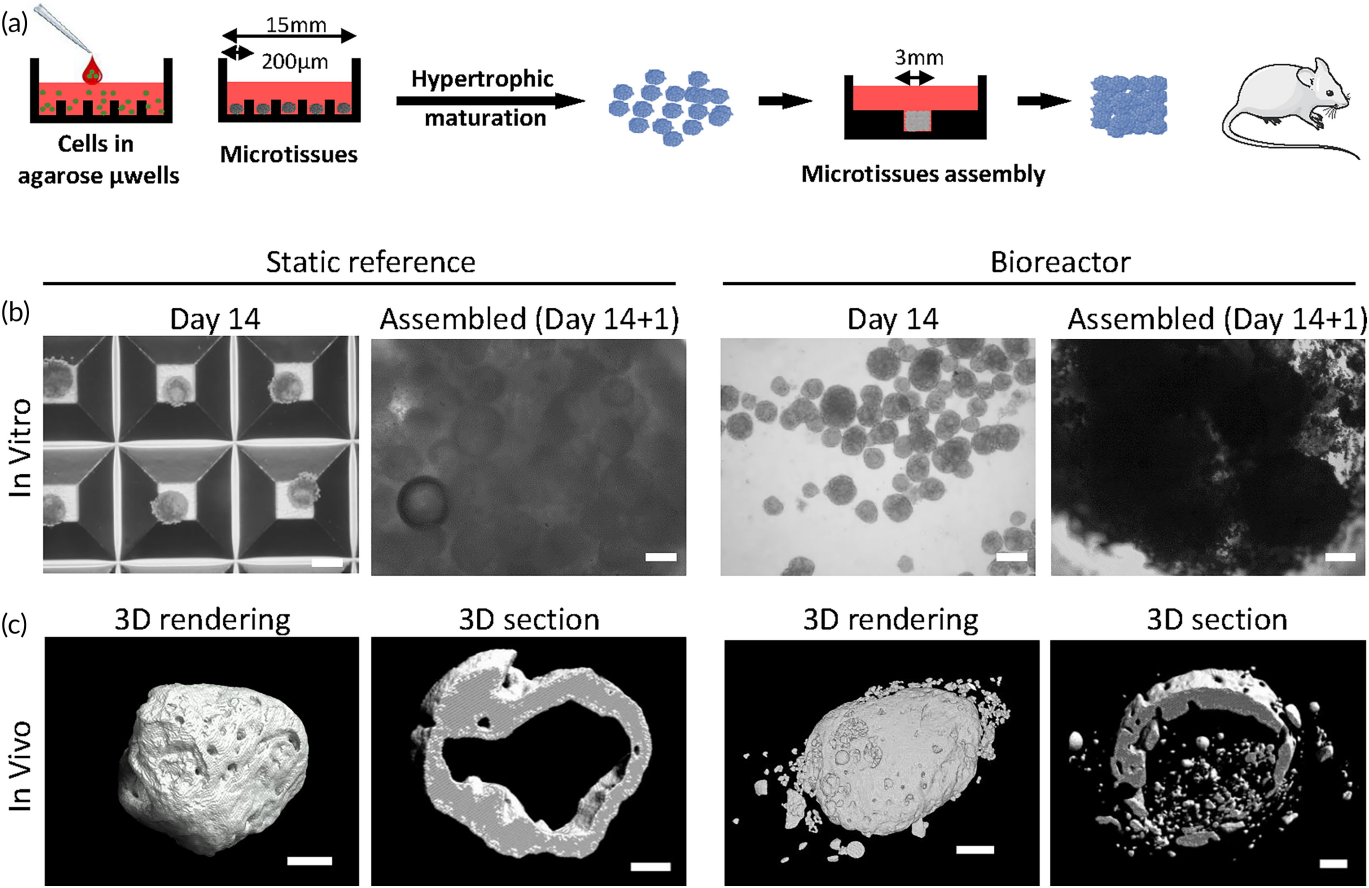
Exometabolomic analysis of medium from hPDCs microtissues cultured in static and in stirred culture analyzed by LC–MS based metabolomics. Extracellular media were sampled at Days 0, 7, and 14. The results were normalized based on DNA content measurements (*n* = 4 individual values ± SEM), *p < 0.05, **p < 0.01, ***p < 0.001, one way ANOVA followed by Tukey's multiple comparison test. Blue color represents the dynamic condition, pink the static large aggregates and red the static microtissues. Impeller speed is 13 rad/s.

We used LC–MS based metabolomics to measure extracellular metabolites in the media of bioreactor‐cultured and in static culture spheroids (Figure 8). Time points of interest were Day 0 (starting of the bioreactor culture), Days 7 and 14. Like the transcriptome level, we saw different metabolic profiles between static and dynamic conditions. More specifically, whereas both conditions were characterized by high glucose consumption and lactate production on Days 7 and 14, in the bioreactor condition there was a significant increase in glucose consumption and lactate production for Day 14. On Day 7 no statistical difference for glucose consumption was observed whereas there was significant difference in lactate production between static and dynamically cultured microtissues. Moreover, the amino acids glutamate, aspartate and citrate showed significant increase in the dynamic condition compared with the static references at Day 7, whereas no significant difference was observed for Day 14. For both Days 7 and 14 we observed a significant increase in alanine production, which can be linked with the high glycolytic profile of the cartilaginous microtissues as mentioned above. Notably, proline, an important amino acid in the context of extracellular matrix function, showed upregulation in dynamic conditions for both Days 7 and 14 whereas at Day 0 it was consumed. The serine secretome profile was distinct in dynamic conditions and the bigger aggregates than the static condition. In the static condition serine was constantly consumed whereas in the dynamic condition and the bigger aggregates serine was in excess in the spent medium. Additionally, serine showed a significant increase in dynamically cultured condition compared with the static large microtissues at both Days 7 and 14.

## 4 DISCUSSION

Cartilage microtissues are promising tissue modules for bottom up biofabrication of implants leading to bone defect regeneration.^29^ Cartilage microtissue differentiation in well‐defined culture conditions recapitulates developmental events encountered in the developing embryonic limb bud which serves as a robust biological paradigm, called developmental engineering.^4^, ^8^, ^29^ Most of the protocols for the development of these cartilaginous microtissues have been carried out in static setups so far, however, a bioreactor‐based suspension process can provide the basis for further scale up as well as automation. In the present study, we explored the impact of dynamic culture in cartilaginous microtissues properties in a novel microbioreactor system generating a stirred environment. However, the dynamic bioprocess in a stirred environment can induce additional maturation cues through mechanical stimulation.

Due to the developmental engineering approach followed in this work, we strived to obtain information from the mechanobiology in embryonic systems. In a seminal study, Carter and Wong^30^ indicated that high shear stresses (in relation to their distribution within the joint) were calculated at the ossific nucleus and at the location where the ossification grove (or zone of Ranvier) was formed. In addition, it was suggested that intermittently applied shear stresses promoted hypertrophic differentiation and endochondral ossification.^30^ As observed by our mathematical modeling study, cartilaginous microtissues were intermittently exposed to regions of high shear stress and low shear stress regimes within the bioreactor system. The distinct difference in proliferation between static and bioreactor conditions could be linked to the commitment of cells towards ECM production rather than proliferation something that is known during chondrogenic differentiation^31^ but in this instance accentuated by the dynamic culture conditions. Additionally, **t**he lower cell density in the bioreactor cultured microtissues (Figure S2) indicates also the commitment of cells towards ECM production in dynamic culture conditions. Studies performed in limb development during embryogenesis revealed that *COLX* and *IHH* expression patterns correlated with stage‐matched patterns of biophysical stimuli,^32^ indicating the importance of these genes as downstream mechano‐transducing genes regulating endochondral ossification. A significant upregulation of both these genes was also observed in our work for the dynamically cultured microtissues when compared with the two static controls, corroborating these basic biology observations in an in vitro context. CNMD is an endogenously anti‐angiogenic protein^33^ that is expressed in the prehypertrophic zone of the growth plate^34^ and in this study was used as an “intermediate” maturity marker. This gene and its upregulation could indicate that microtissue differentiation has reached a level comparable to that of the prehypertrophic zone (given that hypertrophic markers such as COLX and IHH are also clearly upregulated). Furthermore, in a recent study the response to dynamic loading showed enhanced chondrogenic differentiation through upregulation of *COL2A1* and AGGRECAN gene expression in engineered cartilage tissues formed from human MSCs.^35^ Finally, it is interesting to note that in a recent study cartilaginous implants in segmental bone defects resulted in quicker healing when they were mechanically stimulated indicating the crucial role of stresses in the progression of endochondral ossification.^2^ In vitro quality controls indicate that shear stress enhances chondrogenic differentiation of human progenitor cells cultured in in vitro engineered tissues towards a (pre)hypertrophic phenotype. More specifically distinct upregulation of the hypertrophic markers Indian Hedgehog signaling molecule (IHH) and collagen type X alpha 1 chain (COL10A1) together with the significant downregulation of Osterix (OSX) osteogenic marker suggests that the dynamic microtissues showed a later hypertrophic stage than the static ones and thus are more committed to hypertrophy rather than osteogenic differentiation. In vivo implantations demonstrate that microtissues cultured in dynamic conditions are functional and able to undergo endochondral ossification upon implantation. However, they do not show difference to the static condition for the period of implantation. Given that a cortex and a cavity was already present in the explants, in future studies an earlier time point should be examined to assess whether dynamically cultured microtissue could form bone faster than statically cultured ones.

A challenge when trying to study the impact of dynamic culture conditions on cultured cells and tissue modules is the quantification of cell or tissue related mechanical stimuli. In addition, microtissue fusion during culture is another factor that might influence the dispersion within the bioreactor. In order to provide a quantitative link between the cultured microtissues and the process environment, CFD studies only taking into account the development of mechanical stimulus in a continuous medium^36^, ^37^, ^38^ fail to match the actual tissue‐relevant stimulus magnitudes or stimulus history. In this study, we provided a quantitative link between the cultured microtissue suspension and the process environment via coupled CFD‐DEM approach, quantifying mechanical stress at the level of individual microtissues.

There are several recent studies underlying the importance of studying metabolism as a critical regulator for bone regeneration process. Chondrocyte metabolism and more specifically glutamine metabolism controls the collagen synthesis and modification^39^ and targeting skeletal metabolism has been shown as advantageous for the expansion and self‐renewal of skeletal progenitors.^40^ In this study, we aim for exometabolomic analysis, as a first effort to illustrate changes in the metabolome in the dynamic culture and due to the mechanical stimulation. From a metabolic perspective, cartilaginous microtissues showed increased glucose consumption and lactate production during culture time, indicative of a highly glycolytic metabolism which is a characteristic of growth plate chondrocytes.^41^, ^42^ Higher glycolysis has been also reported in chondrocytes under compression.^43^ Additionally, lactate accumulation that is higher in the dynamic condition can be beneficial for bone regeneration. Lactate has been observed to stimulate collagen deposition in an autocrine and paracrine manner, thereby contributing to soft callus progression,^44^ although further in vivo studies will be required to corroborate this for the specific type of microtissue implants. Regarding the amino acid profiles, among the most interesting differences in the context of chondrogenic differentiation, between static and dynamic conditions was proline secretion, which can be linked with the process of extracellular matrix remodeling as seen for similar chondrogenic differentiation processes.^4^ Proline, as a product of ECM degradation can be used as a precursor of other amino acids and as an energy source.^45^, ^46^ Moreover, mechanical stimulation in the bioreactor condition can stimulate the synthesis of matrix degrading enzymes.^47^ Interestingly, we observed aspartate and glutamate secretion significantly higher in the dynamic conditions especially in Day 7. These secretome profiles can be linked with hypertrophic‐like biology, as aspartate is shown to be elevated in synovial fluids from OA patients^48^ whereas aspartate release significantly increases by 90% in IL‐1β stimulated chondrocytes.^49^ The serine secretome profile also showed distinct differences between static and dynamic conditions. Taken together, these metabolites provide a panel of metrics that could be measured from the supernatant providing a target for non‐destructive assessment of the progression of chondrogenic differentiation towards hypertrophy. These secreted metabolites could in the future provide markers that could be monitored during bioreactor culture for non‐destructive assessment of phenotypic state of the cultured microtissues, however further validation also through in vivo studies, is required.

## 5 CONCLUSIONS

In this work, we investigated the impact of dynamic culture conditions during cartilaginous differentiation in microtissues using a novel microbioreactor system. Through the use of mathematical models an in‐silico representation and quantification of the dynamic system and microtissue shear history was conducted. We identified operating conditions where suspension conditions could be maintained and chondrogenic differentiation was possible. This dynamic process led to an accelerated differentiation of cartilaginous microtissues towards hypertrophy that also resulted in distinct metabolic readouts in the culture medium.

## AUTHOR CONTRIBUTIONS

**Niki Loverdou:** Conceptualization (supporting); data curation (lead); formal analysis (lead); funding acquisition (supporting); investigation (lead); project administration (supporting); software (supporting); supervision (supporting); validation (equal); visualization (lead); writing – original draft (lead); writing – review and editing (lead). **Maxim Cuvelier:** Data curation (supporting); formal analysis (supporting); investigation (supporting); software (lead); visualization (supporting); writing – review and editing (supporting). **Gabriella Nilsson Hall:** Data curation (supporting); formal analysis (supporting); investigation (supporting); project administration (supporting); visualization (supporting); writing – review and editing (supporting). **An‐Sofie Christiaens:** Data curation (supporting); formal analysis (supporting); investigation (supporting); visualization (supporting). **Isaak Decoene:** Formal analysis (supporting); visualization (supporting). **Kristel Bernaerts:** Project administration (supporting); supervision (supporting). **Bart Smeets:** Methodology (supporting); project administration (supporting); software (lead); supervision (supporting); writing – review and editing (supporting). **Herman Ramon:** Methodology (supporting); project administration (supporting); supervision (supporting); writing – review and editing (supporting). **Frank P. Luyten:** Supervision (supporting); writing – review and editing (supporting). **Liesbet Geris:** Funding acquisition (supporting); supervision (supporting); writing – review and editing (supporting). **Ioannis Papantoniou:** Conceptualization (lead); funding acquisition (lead); methodology (lead); project administration (lead); resources (lead); supervision (lead); validation (lead); writing – original draft (equal); writing – review and editing (lead).

## CONFLICT OF INTEREST

Gabriella Nilsson Hall is now an employee in Astra Zeneca.

### PEER REVIEW

The peer review history for this article is available at https://publons.com/publon/10.1002/btm2.10468.

## Supporting information


**DATA S1.** Supporting Information

## Data Availability

Data are available on request from the authors
