# Supplementary figures and images for: Stirred culture of cartilaginous microtissues promotes chondrogenic hypertrophy through exposure to intermittent shear stress
Source: Bioeng Transl Med. 2022 Dec 29;8(3):e10468. doi: 10.1002/btm2.10468 (PMC10189438; doi:10.1002/btm2.10468)

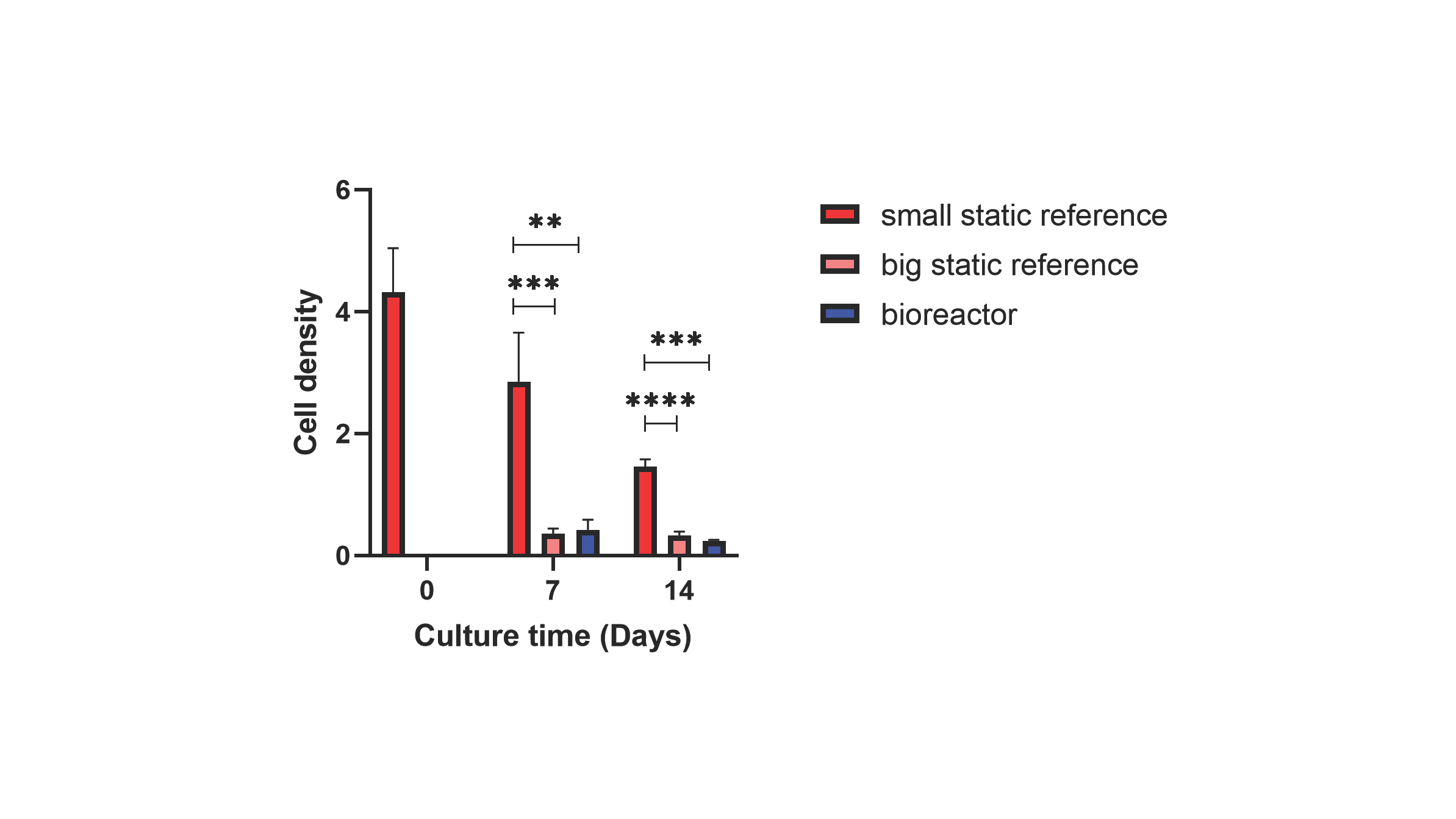

Supplement: Supplementary file 1 — DATA S1. Supporting Information [file BTM2-8-e10468-s001.zip › BTM2_10468_Supplementary Figure 2_Cell density.png]

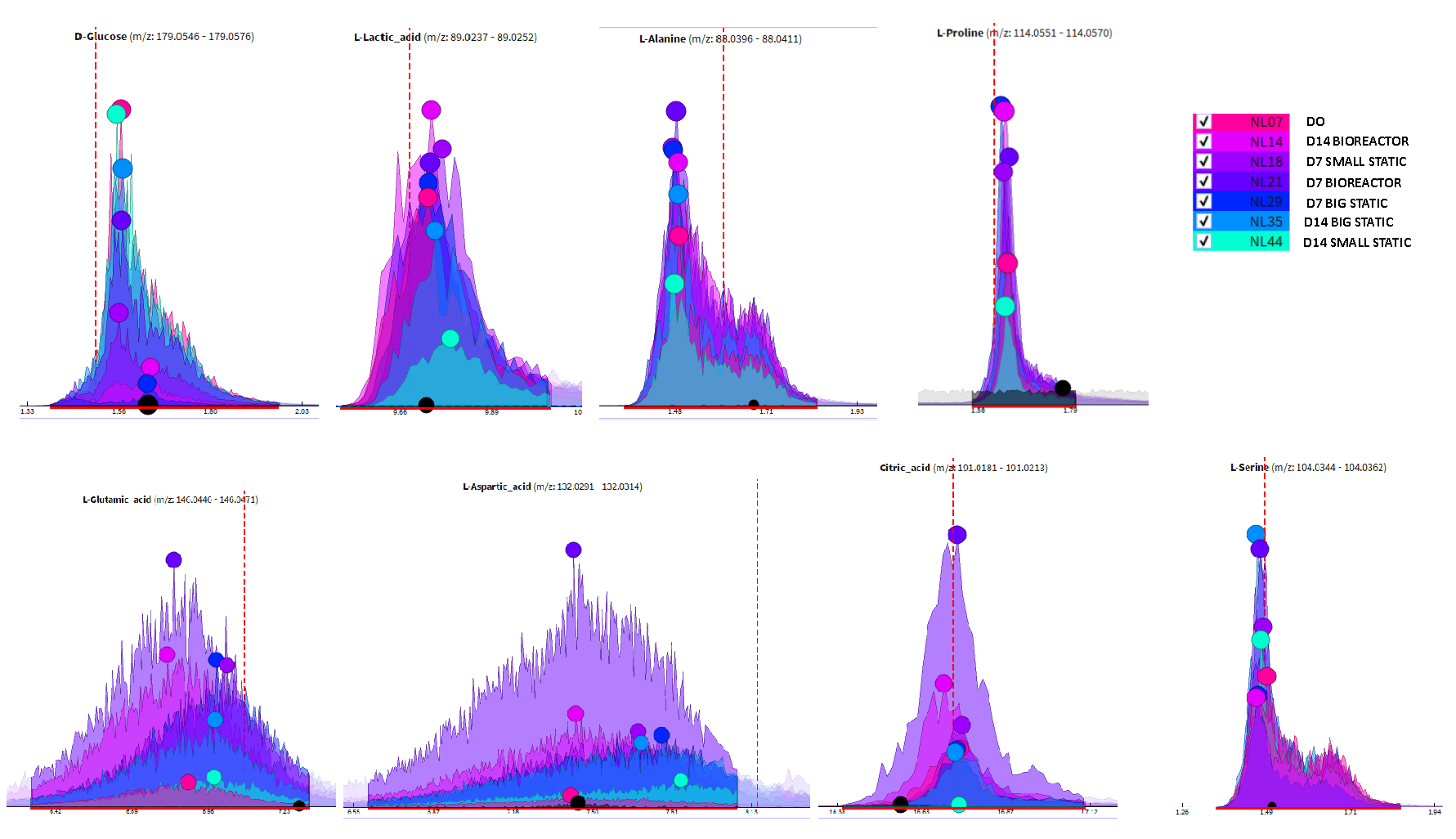

Supplement: Supplementary file 1 — DATA S1. Supporting Information [file BTM2-8-e10468-s001.zip › BTM2_10468_Supplementary Figure 3_Representative Chromatograms_Revision.png]

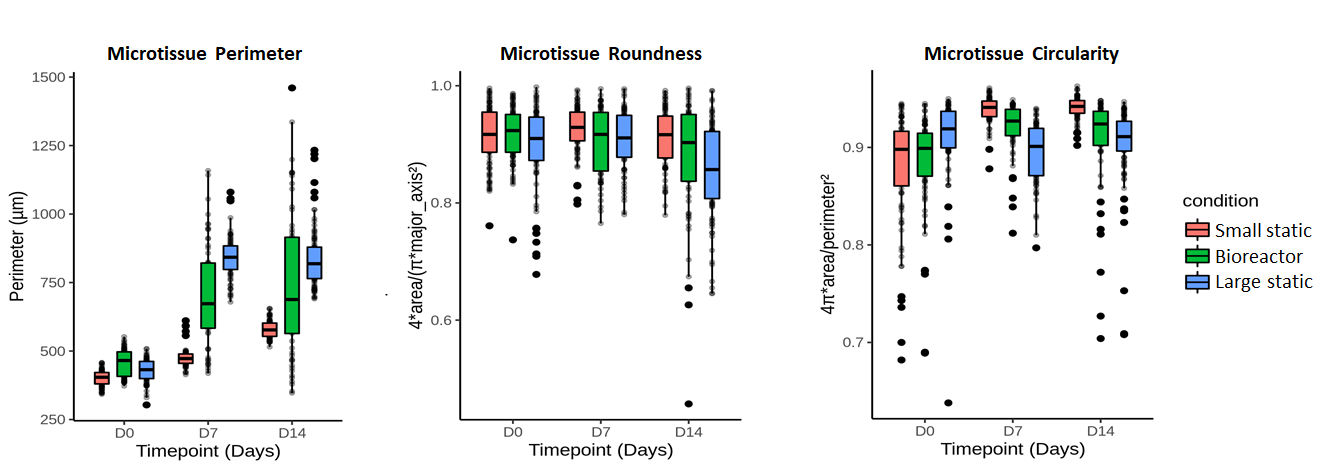

Supplement: Supplementary file 1 — DATA S1. Supporting Information [file BTM2-8-e10468-s001.zip › BTM2_10468_Supplementary Figure_Microtissue Morphology_S2.png]
