# Supplementary material for: Stirred culture of cartilaginous microtissues promotes chondrogenic hypertrophy through exposure to intermittent shear stress
Source: Bioeng Transl Med. 2022 Dec 29;8(3):e10468. doi: 10.1002/btm2.10468 (PMC10189438; doi:10.1002/btm2.10468)
Supplement: Supplementary file 1 — DATA S1. Supporting Information [file BTM2-8-e10468-s001.zip › Supplementary Information_CFD-DEM coupling model.zip!/Supplementary Information_CFD-DEM coupling model/SI_CFD_DEM_coupling model.pdf]

Stirred culture of cartilaginous microtissues promotes chondrogenic hypertrophy through exposure to intermittent shear stress

Loverdou N., Cuvelier M., Nilsson Hall G., Christiaens A.S., Bernaerts K., Smeets B., Ramon, H., Luyten, F.P., Geris, L., Papantoniou I.

Supplementary Note: CFD-DEM coupling model

1 INTRODUCTION

We developed a computational model to characterize fluid-induced shear stress, acting on microtissues in the stirred tank reactor. Similar to the work of Guyot et al., fluid-particle interactions were explicitly modeled via a multi-scale approach [7]. However, in this study we focused on characterizing the distribution of mechanical stimuli acting on a population of microtissues, instead of obtaining high resolution spacial information of the distribution on the surface of individual cells. As such, we used an alternative computational description where microtissues as a whole were treated as individual spherical particles, rather than using an individual cortical shell model for every cell [4]. This significantly reduces the computational effort, albeit at the cost of spatial information.

A Lagrangian approach was used to solve microtissue equation of motion, while fluid equations were solved on a Eulerian grid. To account for particle-fluid interaction forces we used a point-coupled CFD-DEM approach, where data from the Lagrangian particles were projected on the Eulerian grid and vice-versa [2]. The specific methodology and order of the coupling typically depends on the particle-fluid volume ratio, Reynolds and Stokes number, all of which are discussed in-depth in section 2.3.

2 GOVERNING EQUATIONS

Simulating particles in a stirred tank reactor requires an accurate mechanical description of both particle-, and fluid motion. To resolve the fluid flow in the mini-bioreactor, we used a Finite Volume Method (FVM) to solve the Navier-Stokes equations on a Eulerian grid. For the microtissues, we opted to use a lattice-free Lagrangian description, as we were interested in the discrete trajectories of individual particles. Choosing the Discrete Element Method (DEM), where individual microtissues are represented as deformable spheres in a center-based model (CBM), allowed us to formulate an equation of motion for each microtissue, where we explicitly took particle-particle interaction forces into account [10]. Furthermore, modeling the microtissues using a DEM approach allows for the quantification of relevant mechanical properties, at an individual level. In contrast to bulk (Eulerian) approaches, this coupled Lagrangian-Eulerian approach takes into account the history of each individual particle, providing more accurate estimates for relevant mechanical output measures, when compared to e.g. bulk multi-phase CFD approaches [5, 9].

2.1 Fluid phase equations of motion

The fluid in the mini-bioreactor was assumed to be incompressible and taking into account particle-fluid interactions, the Navier-Stokes equations could be rewritten as

$$\frac{\mathrm{d}\alpha}{\mathrm{d}t} + \nabla \cdot \alpha U = 0 \tag{1}$$

$$\rho_f \left(\frac{\mathrm{d}\alpha U}{\mathrm{d}t} + (\alpha U \cdot \nabla) U \right) = -\alpha \nabla P + \mu_f \nabla^2 \alpha U + M_e, \tag{2}$$

assuming constant fluid density ρ_f and viscosity μ_f. M_e is the momentum exchange between the fluid and particles, α corrects the local free volume of the CFD cells for the presence of DEM particles. The specific values of M_e and α, depend on the order of the CFD-DEM coupling, see section 2.3. Finally, for setups in which the Reynolds number for a stirred vessel is low,

$$\mathrm{Re}_f = \frac{4\rho_f \Omega R_i^2}{\mu_f} < 2000$$

with Ω the rotational velocity and R_i the radius of the impeller, inertial contributions (left hand side of eq. 2) are negligible, collapsing the Navier-Stokes equations to the (diffusion dominated) Stokes equations. The fluid phase equations were solved using the OpenFOAM framework via a custom implemented solver, which was based on the icoFOAM algorithm [1]. No-slip boundary conditions were assumed for rotor and stator, while slip conditions were assumed at the liquid-air interface. Symmetry boundary conditions could not be used, as the distributions of M_e and α depend on particle trajectories. The Eulerian grid representation of the fluid domain was based on engineering drawings of the mini-bioreactor and were generated with the blockMesh and snappyHexMesh algorithms [6].

2.2 Equation of motion of the particles

In the CBM, individual microtissues are represented by deformable spherical particles with radius R based on experimentally measured size distributions of microtissues cultured in mini-bioreactors. Given the CBM, we solve an equation of motion for each particle center of mass x, to simulate how the microtissues move and interact. For this, we explicitly determine the forces acting on individual particles at each time step [10, 11]. The methodology presented in this work was based on the work of Smeets et al. albeit, expanded to explicitly account for particle-fluid interaction forces, see section 2.3 [10] We start by providing a general overview before addressing the individual forces acting on the particles. Assuming Stokes drag, the particle-fluid slip velocity can be estimated by the terminal velocity

$$v_s = \frac{2}{9}\frac{\Delta\rho_f g R^2}{\mu_f}$$

for a free floating particle settling in a gravitational field. This gives us an estimate for the particle Reynolds number

$$\mathrm{Re}_p = \frac{2\left\|v_s\right\|\rho_f R}{\mu_f} < 1,$$

which confirms friction forces to be dominant over inertial forces. This allowed us to formulate an over-damped equation of motion for each particle i

$$F_{g,i} + F_{e,i} + \sum_j F_{c,ij} + \sum_j F_{f,ij} = 0, \tag{3}$$

where we identified gravitational F_g, fluid-particle F_e, contact F_c and friction F_f force contributions, acting on particle i in contact with particles j. Splitting $F_e = F_{e'} + \Gamma_{ii}\left(v_f - v_i\right)$ into velocity dependent and independent contributions, and substituting $F_{f,ij} = \Gamma_{ij}\left(v_j - v_i\right)$, eq. 3 could be rewritten in function of particle center of mass velocities v_i

$$F_{g,i} + F_{e',i} + \Gamma_{ii} v_f + \sum_j F_{c,ij} = \Gamma_{ii} v_i + \sum_j \Gamma_{ij}(v_i - v_j). \tag{4}$$

For a system consisting of n particles we get

$$\mathbf{F} = \Gamma \mathbf{V}, \tag{5}$$

where all velocity-independent forces, the left hand side in eq. 4, are captured column matrix $\mathbf{F}$ with dimensions $3n \times 1$. Friction matrix Γ, with dimensions $3n \times 3n$, is positive definite and, given the sparse connectivity of the system, diagonally dominant. This allows us to iteratively solve the system for $\mathbf{V}$, via the conjugate gradient method. Particle center of mass positions $\mathbf{X}$ could then be updated via an explicit Euler scheme

$$\mathbf{X}_{t+\Delta t,i} = \mathbf{X}_{t,i} + \mathbf{V}_{t+\Delta t,i}\Delta t, \tag{6}$$

with time-step Δt.

2.3 Contact and friction forces

During culturing in the mini-bioreactor, microtissues can collide with each other and components of the mini-bioreactor (e.g. stator and rotor). To model these interactions and estimate contact forces, we assumed the microtissues to behave as deformable elastic bodies and used the Johnson-Kendall-Roberts (JKR) approximation to describe contact interactions. [3, 8] The JKR contact model assumes an indentation-dependent contact force, which can be split into a purely elastic (repulsive) and adhesive (attractive) contribution. The repulsive force is based on a Hertzian force with magnitude

$$F_{r,ij} = \frac{4\hat{E}_{ij}}{3\hat{R}_{ij}} a_{ij}^3,$$

given contact stiffness $\hat{E}_{ij} = \left(\frac{1-\nu_i^2}{E_i} + \frac{1-\nu_j^2}{E_j}\right)^{-1}$ and contact radius $\hat{R}_{ij} = \left(\frac{1}{R_i} + \frac{1}{R_j}\right)$. The magnitude thus scales with the Young's moduli E (Pa), Poisson numbers ν $(-)$, radii R and contact area a of particles i and j. The magnitude of adhesive contributions

$$F_{a,ij} = -\sqrt{8\omega_{ij}\hat{E}a_{ij}^3},$$

scale with adhesive energy density ω_{ij} (J/m^2) of the contact pair ij. Contact area a_{ij} is obtained by iteratively solving for a,

$$\delta = \frac{a^2}{\hat{R}} - \sqrt{\frac{2\pi\omega}{\hat{E}}a},$$

given overlap distance δ. The total contact force, $F_{c,ij} = (F_{a,ij} + F_{r,ij})n_{ij}$, is applied in the normal contact direction n_{ij}.

Particles in contact also exert friction forces on each other based on their relative velocities. The contact friction tensor is formulated as

$$\Gamma_{ij} = a_{ij}\left[\gamma_n n_{ij} \otimes n_{ij} + \gamma_t\left(\mathbb{I} - n_{ij} \otimes n_{ij}\right)\right],$$

where a distinction is made between normal γ_n and tangential γ_t contributions and $\otimes$ denotes the outer product. The friction force acting on particle i due to contact ij is then give by $\Gamma_{ij}(v_j - v_i)$.

2.4 Particle-fluid interaction forces

To estimate fluid-particle interaction forces, we used our estimates for the particle Reynolds number Re_p, and Stokes number $\mathrm{Stk} = \tau_p/\tau_f$, with

$$\tau_p = \frac{2\rho_p R^2}{9\mu_f}$$

and $\tau_f \approx 2R/v_0$, to determine which forces should be taken into account. As estimated Re_p were smaller than unity, inertial contributions were assumed to be negligible. Furthermore, given the low Stk, particles were assumed to follow the streamlines of the fluid accurately. As the microtissues were smaller than the estimated fluid length scale, a point-force coupling was used, where all hydrodynamic interaction forces were represented as point forces acting on the center of mass of the submerged particles. As we do not resolve particle rotations, Magnus effect and Saffman lift forces were neglected. Lift forces due to the pressure gradient and fluid shear are however accounted for. The total fluid-particle interaction force is then given by

$$F_e = F_d + F_l \tag{7}$$

with fluid-particle drag force $F_d = 6\pi\mu_f R v_s = \Gamma v_s$ and fluid-particle lift force

$$F_l = \frac{4\pi R^3}{3}(-\nabla P + \nabla \cdot \tau).$$

It should be noted that the buoyancy force due to the displaced fluid volume is accounted for in the pressure lift contribution. The interaction force is applied to the particles while a reaction force is applied to the fluid.

3 CFD-DEM COUPLING

For a four-way coupling, the momentum exchange between a particle and the fluid can be estimated as

$$M_e = \sum -\frac{g(\|x - x_p\|)F_e}{\alpha V}$$

with fluid-particle interaction force F_e. However, given the low Stk and Re, we opted to us a one-way coupling where $M_e = 0$. The free volume estimator

$$\alpha(x) = \sum_p g(\|x - x_p\|)\frac{V - V_p}{V}$$

corrects the local volume of the CFD cells for the presence of DEM particles with volumes V_p.

A numerical smoothing function $g(r)$ is used to interpolate between the Lagrangian (DEM) particles and the Eulerian (CDF) grid. It is characterized by a length-scale σ, monotonically decreasing on $\mathbb{R}_+$, and normalized so that

$$\int_{\mathbb{R}^3} g(r)\,\mathrm{d}x = 1. \tag{8}$$

For this work, a Gaussian kernel

$$g(r) = \frac{1}{(2\pi\sigma^2)^{3/2}} \exp\left(\frac{-r^2}{2\sigma^2}\right)$$

was used, with $\sigma = L/\sqrt{8\ln(2)}$, where L represents the full width at half maximum. We opted to set $L = 6\hat{R}$ for setups with mean particle radius $\hat{R}$. However, instead of solving $g(r)$ for all particles on every point in the Eulerian grid, we approximated the smoothed fields by solving the diffusion equation

$$\sum_p g(\|x - x_p\|) I_p \approx I(x,t') + D\nabla^2 I(x,t')\mathrm{d}t' \tag{9}$$

for one time-step, where I_p represents a particle variable that needs to be transferred to the Eulerian grid and $I(x,t')$ the grid approximation where

$$I(x,t') = \begin{cases} I_p\delta(x_p) & \forall x_p, \\ 0 \end{cases}. \tag{10}$$

To ensure that this approximation is valid, the diffusion coefficient D needs to be set as

$$D = \frac{L^2}{16\ln(2)\mathrm{d}t'},$$

given time step $\mathrm{d}t'$. It should also be noted that the projection error in eq. 10, due to the spatial discretization of the Eulerian grid, decreases with smaller CFD cell sizes.

4 STRESS ESTIMATES

To quantify mechanical stress caused by the dynamic culturing conditions on the micro-reactor, we need to estimate the velocity gradient over the individual particles. Multiplying the gradient with the fluid viscosity, we get an estimator for Stokes' stress tensor

$$\tau = \mu_f\left(\nabla v + (\nabla v)^T\right). \tag{11}$$

The mean stress due to fluid-particle interactions, acting on a given particle is estimated by the Frobenius norm of Stokes' stress tensor

$$\sigma_F = \sqrt{\sum_i^m \sum_j^n \mid \tau_{ij} \mid^2}. \tag{12}$$

Similarly, the maximum shear stress can then be estimated by calculating the Von Mises stress or equivalent tensile stress

$$\sigma_{VM} = \sqrt{\frac{(\tau_{11}-\tau_{22})^2 + (\tau_{22}-\tau_{33})^2 + (\tau_{33}-\tau_{11})^2 + 6\left(\tau_{12}^2 + \tau_{23}^2 + \tau_{31}^2\right)}{2}}, \tag{13}$$

from Stokes' stress tensor.

5 STEADY-STATE ANALYSIS

The experimental mini-bioreactor setup was recreated *in-silico*, where a seeding density of $1 \times 10^6\,\mathrm{L}^{-1}$ microtissues was used to initialize the simulations, resulting in roughly 2000 microtissues per simulated bioreactor. Microtissues are modeled as spheres with a radius drawn from a normal distribution, see Table 2. During the simulations, mechanical and geometric variables such as (relative) particle position, fluid-particle slip velocity and fluid-induced stress, were probed at microtissue level to characterize the represent the mechanical micro-environment. As the reactors reached a steady-state, mean-temporal variable values were calculated per particle over a period of 30 seconds. In this work, three impeller velocities Ω were tested, mimicking three distinct shear conditions: low $(7\,\mathrm{rad\,s^{-1}})$, medium $(13\,\mathrm{rad\,s^{-1}})$ and high $(20\,\mathrm{rad\,s^{-1}})$.

Table 1 Estimated sediment size (in number of simulated microtissues) as a function of impeller velocity Ω

Label	Ω $(\mathrm{rad\,s^{-1}})$	Sediment size (number of particles)
Low	7	904 ± 5
Medium	13	290 ± 3
High	20	3 ± 1

For all setups, two distinct populations could be observed. A low-shear population which exists as sediment in the reactor dead zone, and a suspended high-shear population, see main text. The relative size of both populations varies with the rotation velocity. For low rotation velocities, the low-shear population dominates. As the rotation velocity increases, the suspended population becomes more prevalent, see table 1.

6 EFFECT OF MICROTISSUE SIZE

In order to investigate numerically the effect of microtissue size on the distribution of spheroids in the bioreactor and on the experienced shear stress, we performed a separate set of simulations at $\Omega = 20\mathrm{rad\,s^{-1}}$, where we compare two microtissue sizes: "Small", of 50 µm radius, and "Large" of 100 µm radius. Moreover, we compare these two cases in three separate tissue spheroid density regimes: neutral buoyancy, low relative particle density and high relative particle density. The results of these simulations are summarized in Fig. 1. The average height of the particles in steady-state demonstrates that for high relative density, a distinct size segregation could occur, with sedimentation occurring for the larger microtissues whereas the smaller microtissues remain suspended. This renders the effect of different microtissue size on the shear stress an intricate phenomenon. On the one hand, the larger velocity gradient on larger particles implies a larger shear stress, an effect that can be clearly seen on the top row of Fig. 1, for neutral particle buoyancy. On the other hand, larger particles are more prone to sediment to the bottom of the bioreactor, where lower velocity gradients are encountered and the experienced shear stress is smaller. The net result of these two effects combined for this setup produces a similar distribution of shear stress between small and large spheroids at low and high relative particle density.

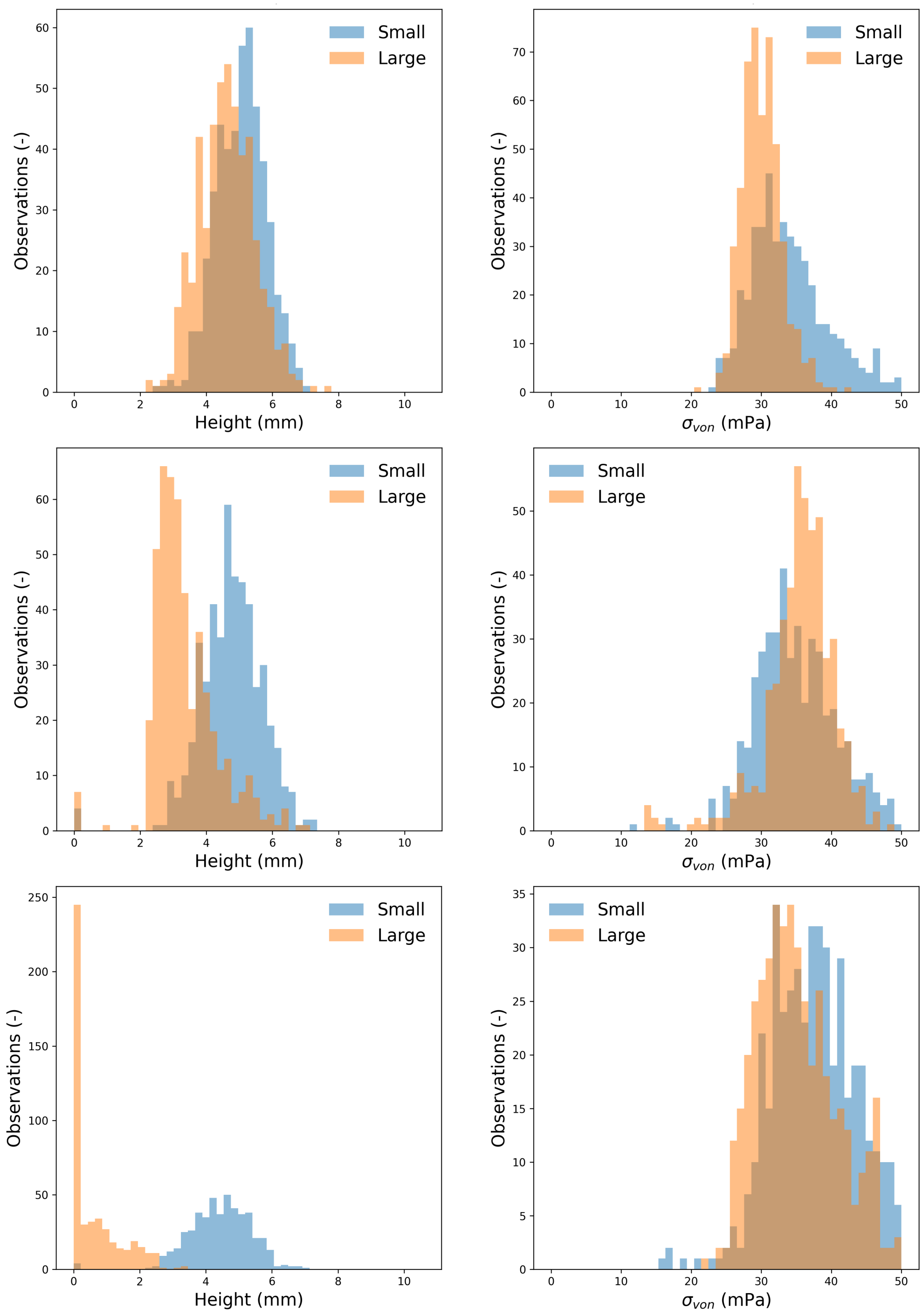

Figure 1 Comparison of steady-state particle height in the bioreactor on the left and experienced shear stress (von Mises stress) on the right between small ($R_p = 50\mu\text{m}$) and large ($R_p = 100\mu\text{m}$), for neutral buoyancy of particles ($\rho_p = 1000\text{kg}/\text{m}^3$, top row), low relative density ($\rho_p = 1025\text{kg}/\text{m}^3$, middle row) and high relative particle density ($\rho_p = 1050\text{kg}/\text{m}^3$, bottom row).

7 SIMULATION PARAMETERS

Table 2 Microtissue properties used in the simulations

**Parameter**	Symbol	Value
Microtissue Radius	R_p	$55 \pm 10\,\mu m$
Young's modulus	E	$600\,Pa$
Poisson's ratio	ν	$1/3$
Adhesion energy density	ω	$0.1\,mJ/m^2$
Particle density	ρ_p	$1010\,kg/m^3$
Normal friction	γ_n	$1\,Pa\,s\,\mu m^{-1}$
Tangential friction	γ_t	$1\,Pa\,s\,\mu m^{-1}$

Table 3 Reactor properties used in the simulations

**Parameter**	Symbol	Value
Impeller radius	R_i	$6.15\,\mu m$
Fluid viscosity	μ_f	$0.999\,mPa\,s^{-1}$
Fluid density	ρ_p	$981\,kg/m^3$
Rotor velocity	Ω	$7\,rad\,s^{-1}$
Reactor volume	V_r	$1.95\,mL$
Seeding density	ρ_S	$1 \times 10^6\,L^{-1}$

REFERENCES

[1] Behrens, T. (2009). Openfoam's basic solvers for linear systems of equations. *Chalmers, Department of Applied Mechanics*, 18(02).

[2] Chen, F., Drumm, E. C., and Guiochon, G. (2011). Coupled discrete element and finite volume solution of two classical soil mechanics problems. *Computers and Geotechnics*, 38(5):638–647.

[3] Chu, Y.-S., Dufour, S., Thiery, J. P., Perez, E., and Pincet, F. (2005). Johnson-kendall-roberts theory applied to living cells. *Physical review letters*, 94(2):028102.

[4] Cuvelier, M., Pešek, J., Papantoniou, I., Ramon, H., and Smeets, B. (2021). Distribution and propagation of mechanical stress in simulated structurally heterogeneous tissue spheroids. *Soft Matter*.

[5] Delafosse, A., Loubière, C., Calvo, S., Toye, D., and Olmos, E. (2018). Solid-liquid suspension of microcarriers in stirred tank bioreactor–experimental and numerical analysis. *Chemical Engineering Science*, 180:52–63.

[6] Gisen, D. (2014). Generation of a 3d mesh using snappyhexmesh featuring anisotropic refinement and near-wall layers. In *ICHE 2014. Proceedings of the 11th International Conference on Hydroscience & Engineering*, pages 983–990.

[7] Guyot, Y., Smeets, B., Odenthal, T., Subramani, R., Luyten, F. P., Ramon, H., Papantoniou, I., and Geris, L. (2016). Immersed boundary models for quantifying flow-induced mechanical stimuli on stem cells seeded on 3d scaffolds in perfusion bioreactors. *PLoS computational biology*, 12(9):e1005108.

[8] Johnson, K. L., Kendall, K., and Roberts, a. (1971). Surface energy and the contact of elastic solids. *Proceedings of the royal society of London. A. mathematical and physical sciences*, 324(1558):301–313.

[9] Qi, N., Zhang, H., Zhang, K., Xu, G., and Yang, Y. (2013). Cfd simulation of particle suspension in a stirred tank. *Particuology*, 11(3):317–326.

[10] Smeets, B., Odenthal, T., Tijskens, E., Ramon, H., and Van Oosterwyck, H. (2013). Quantifying the mechanical micro-environment during three-dimensional cell expansion on microbeads by means of individual cell-based modelling. *Computer methods in biomechanics and biomedical engineering*, 16(10):1071–1084.

[11] Van Liedekerke, P., Palm, M., Jagiella, N., and Drasdo, D. (2015). Simulating tissue mechanics with agent-based models: concepts, perspectives and some novel results. *Computational particle mechanics*, 2(4):401–444.
